# Supplementary material for: Enhanced hexamerization of insulin via assembly pathway rerouting revealed by single particle studies
Source: Commun Biol. 2023 Feb 15;6:178. doi: 10.1038/s42003-022-04386-6 (PMC9932072; doi:10.1038/s42003-022-04386-6)
Supplement: Supplementary file 2 — Supplementary Information [file 42003_2022_4386_MOESM2_ESM.pdf]

Supplementary information

Freja Bohr[1,2], Søren S.-R. Bohr[1,2], Narendra Kumar Mishra[1,#], Nicolás Sebastian González Foutel[3,#], Henrik Dahl Pinholt[1,2,4], Shunliang Wu[1], Emilie Milan Nielsen[1,2], Min Zhang[1,2], Magnus Kjaergaard[3], Knud J. Jensen[1]* & Nikos S. Hatzakis[1,2]*

Affiliations

[1] Department of Chemistry & Nanoscience Center, University of Copenhagen, Copenhagen, Denmark.

[2] Novo Nordisk Foundation Center for Protein Research, Faculty of Health and Medical Sciences, University of Copenhagen, Copenhagen, Denmark.

[3] Department of Molecular Biology and Genetics, The Danish Research Institute for Translational Neuroscience (DANDRITE), Nordic EMBL Partnership for Molecular Medicine, and Center for Proteins in Memory PROMEMO, Danish National Research Foundation, Aarhus University, Aarhus Denmark.

[4] Physics Department, Massachusetts Institute of Technology, Cambridge, Massachusetts 02139.

[#] These authors contributed equally

Corresponding authors

* e-mail kjj@chem.ku.dk; hatzakis@chem.ku.dk

Preprint Servers

BioRXiv: https://www.biorxiv.org/content/10.1101/2022.04.06.487286v1.full

Keywords

Single-molecule fluorescence microcopy, insulin self-assembly, insulin hexamerization, pathway rerouting, quantitative image analysis.

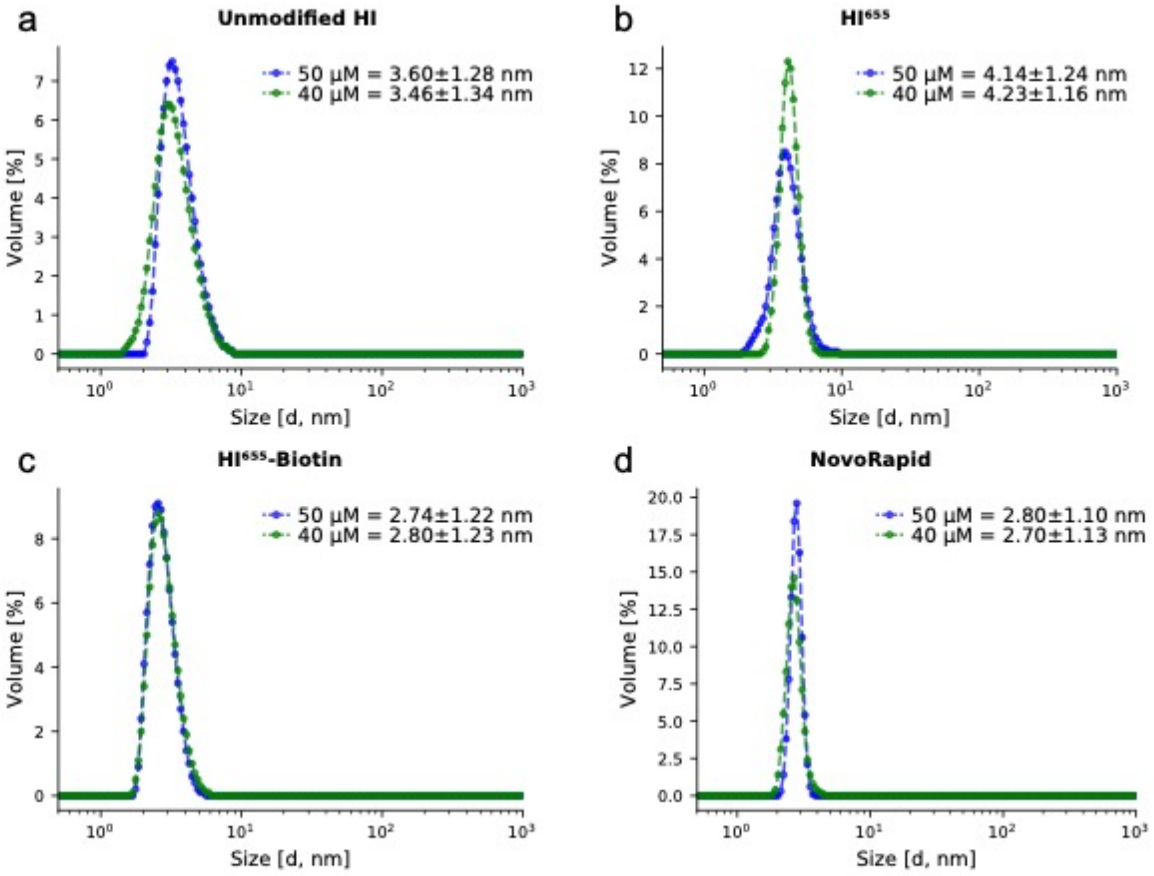

Supplementary Figure 1: Dynamic Light Scattering experiments at varying concentration. **a,** DLS measurements of 50 and 40 µM unmodified HI in 10 mM Na_2HPO_4, 10 mM Na_2HPO_4, 10 mM NaH_2PO4, and 5 mM NaCl at pH 7.5. **b,** DLS measurements of 50 and 40 µM HI^{655} in 10 mM Na_2HPO_4, 10 mM Na_2HPO_4, 10 mM NaH_2PO4, and 5 mM NaCl at pH 7.5. **c,** DLS measurements of 50 and 40 µM HI^{655}-Biotin in 10 mM Na_2HPO_4, 10 mM Na_2HPO_4, 10 mM NaH_2PO4, and 5 mM NaCl at pH 7.5. **d,** DLS measurements of 50 and 40 µM NovoRapid in 10 mM Na_2HPO_4, 10 mM Na_2HPO_4, 10 mM NaH_2PO4, and 5 mM NaCl at pH 7.5.

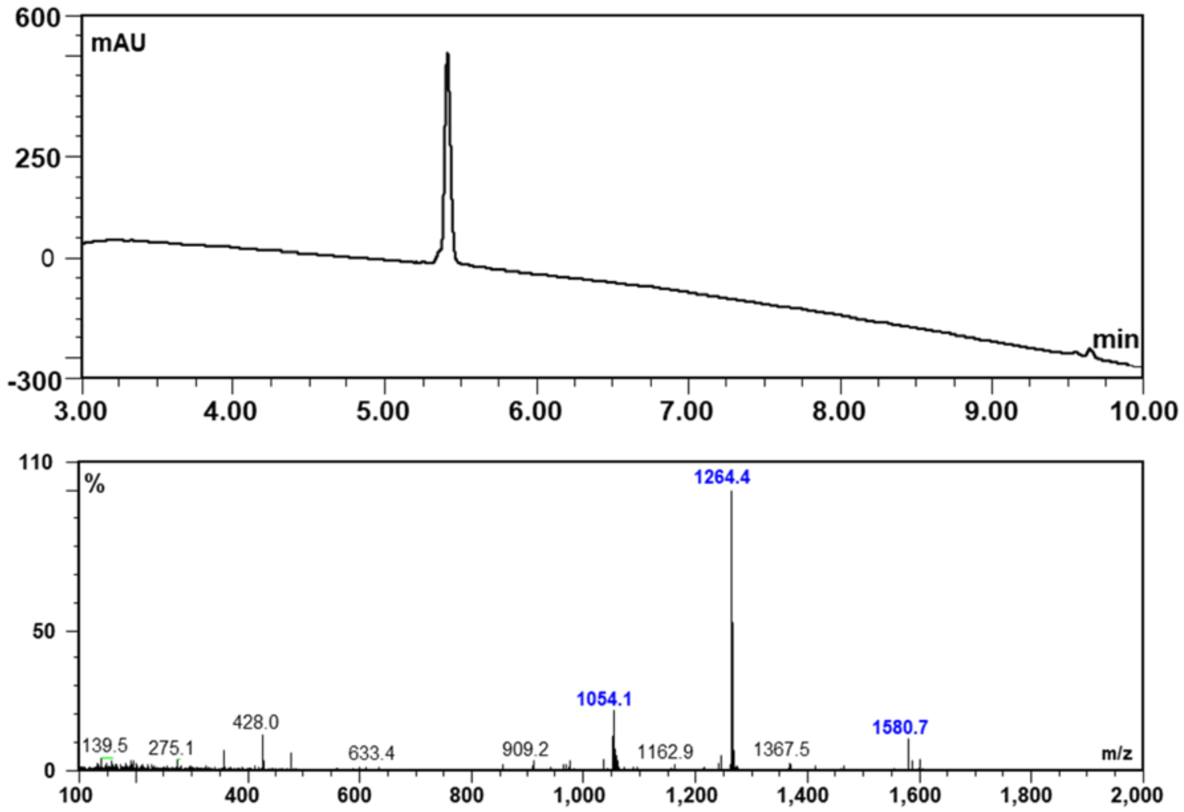

Supplementary Figure 2: LCMS chromatogram and mass spectrum for HI^{655} after purification.

Serial No.	Peak	Calculated	Observed
1.	$[M]^{+}$	6318.26	Not Observed
2.	$[M+4H]^{4+}$	1579.72	1580.7
3.	$[M+5H]^{5+}$	1263.98	1264.4
4.	$[M+6H]^{6+}$	1053.65	1054.1

Supplementary Table 1: LCMS analysis of $HI^{655.}$

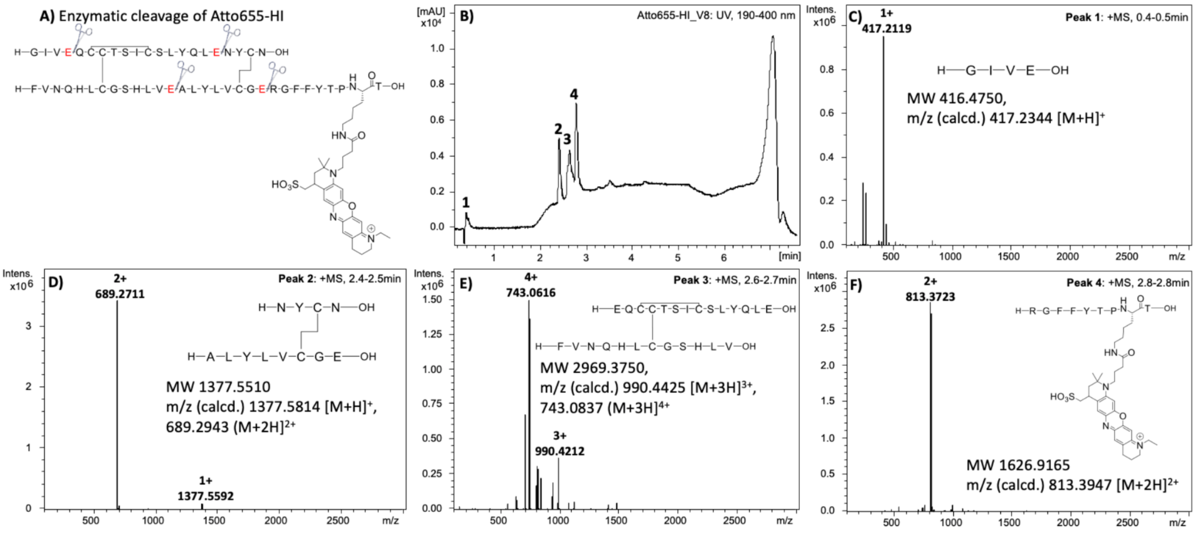

Supplementary Figure 3: Enzymatic cleavage study of pure Atto655-HI using Endoproteinase Glu-C (V8 protease) from *Staphyloccus aureus* V8. **a,** The C-terminal V8 cleavage site at glutamic acid (E) indicated in red. **b,** HPLC (UV) chromatogram of pure Atto655-HI treated with V8 protease. Four peaks were observed and showed in their respective mass spectrum **c,** to **f,**. **f,** The observed mass in peak 4 showed the acylation of Atto655 at the B29 Lysine of HI.

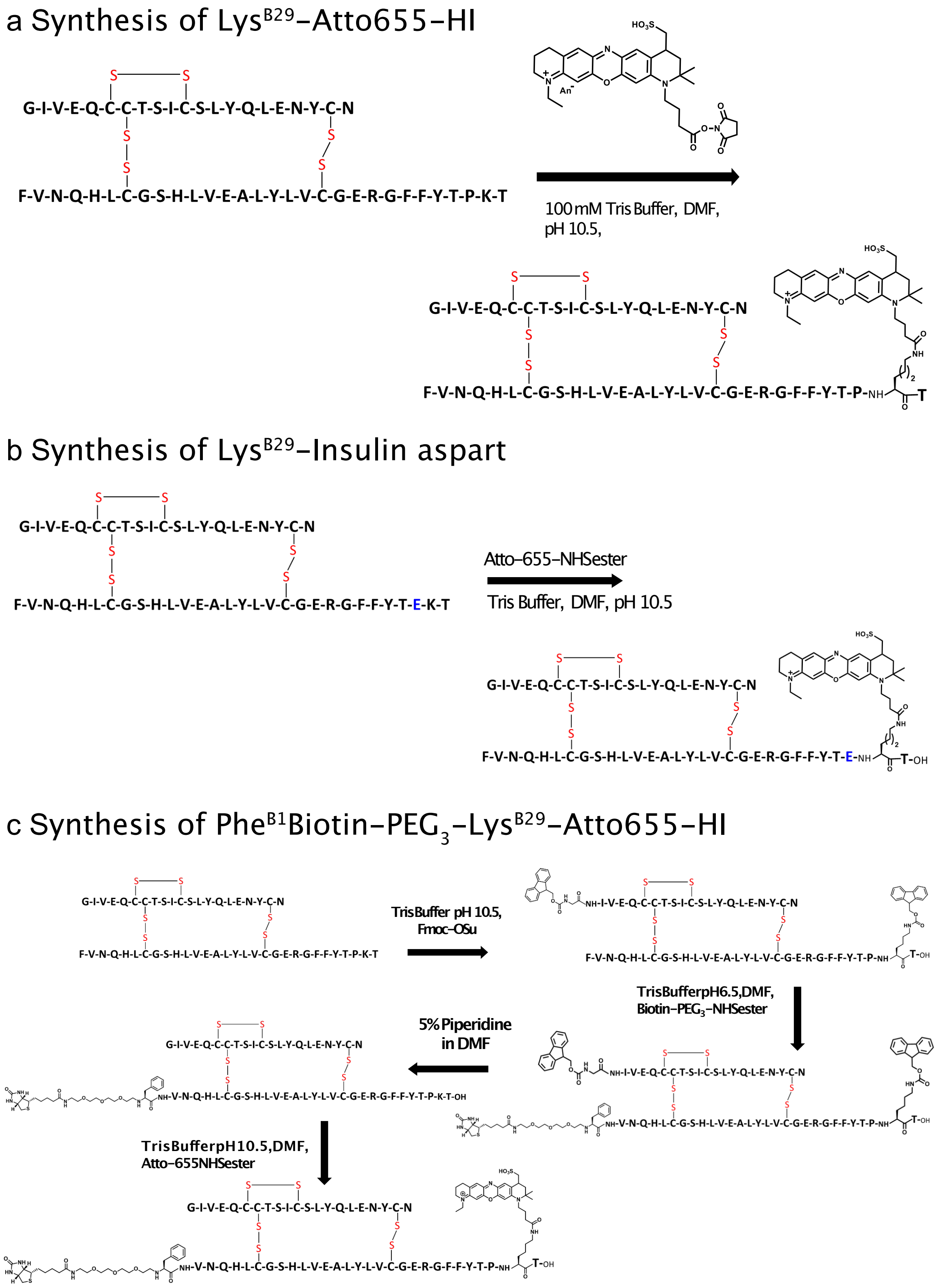

Supplementary Scheme 1: a, For the synthesis of Lys^{B29}-Atto655-HI **b,** For the synthesis of Lys^{B29}-Insulin aspart **c,** For the synthesis of Phe^{B1}Biotin-Lys^{B29}-Atto655-HI

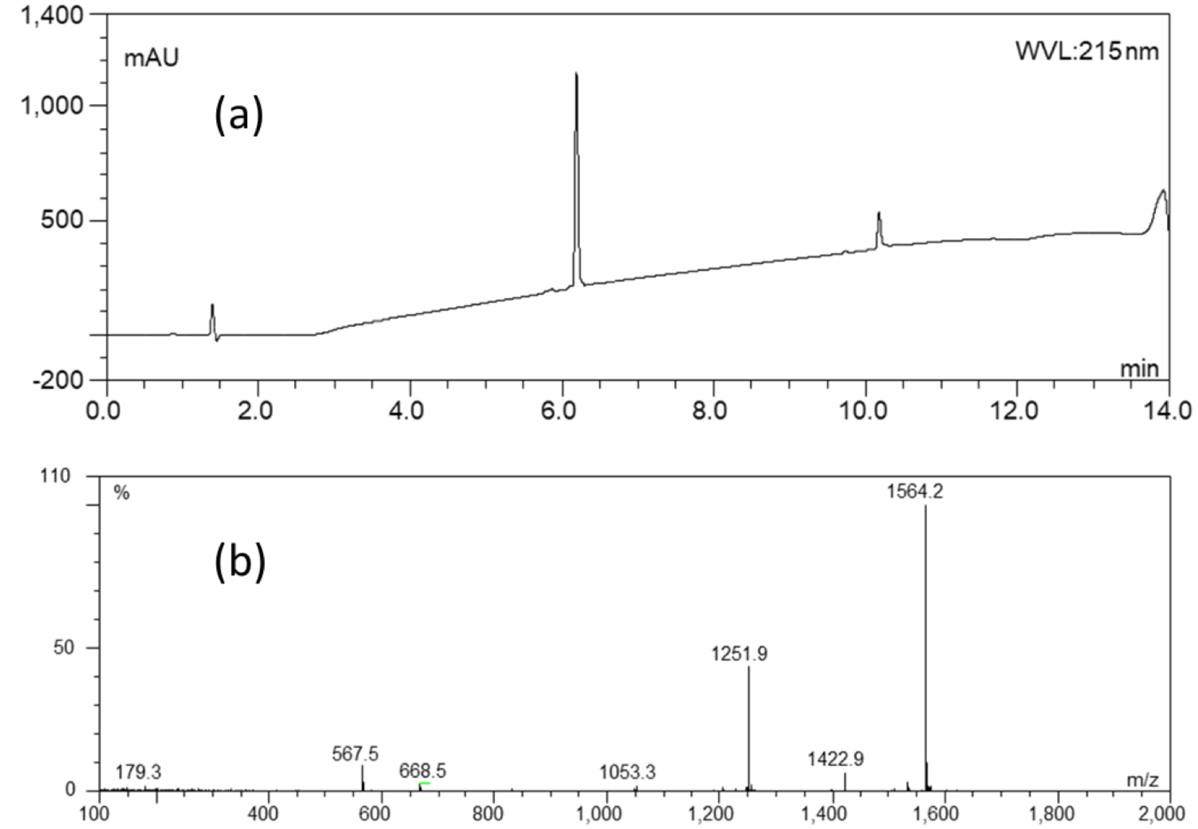

Supplementary Figure 4: After purification **a,** LCMS chromatogram of Di-Fmoc-HI **b,** Mass spectra of Di-Fmoc-HI

Serial No.	Peak	Calculated	Observed
1.	$[M]^+$	6250.78	Not Observed
2.	$[M+4H]^{4+}$	1563.45	1564.2
3.	$[M+5H]^{5+}$	1250.96	1251.9

Supplementary Table 2: LCMS Analysis of Di-Fmoc-HI

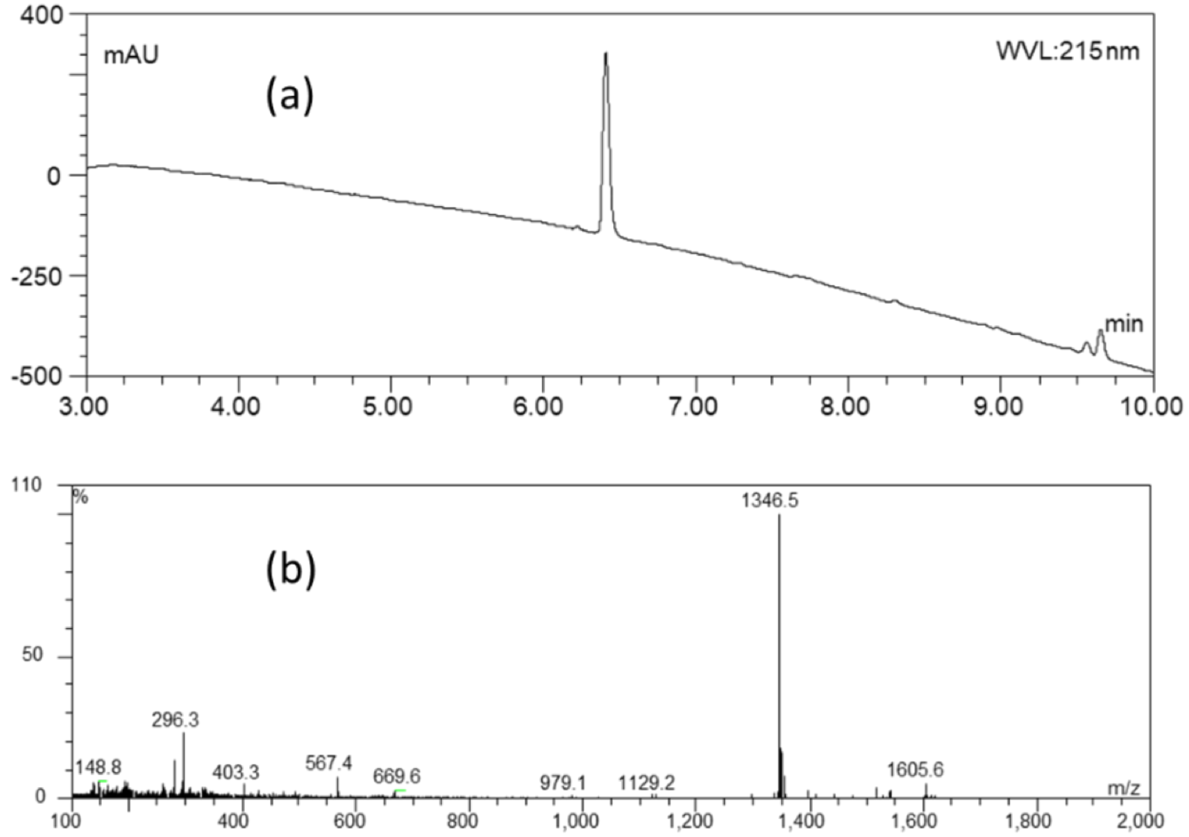

Supplementary Figure 5: a, LCMS chromatogram of DiFmoc-*Phe*^{B1}*Biotin-PEG*$_3$-Human Insulin, **b,** Mass spectra of DiFmoc-Phe^{B1}*Biotin-PEG*$_3$-Human Insulin.

Serial No.	Peak	Calculated	Observed
1.	$[M]^+$	6720.993	Not Observed
2.	$[M+4H]^{4+}$	1345.808	1346.5

Supplementary Table 3: LCMS Analysis of DiFmoc-Phe^{B1}*Biotin-PEG*$_3$-HI

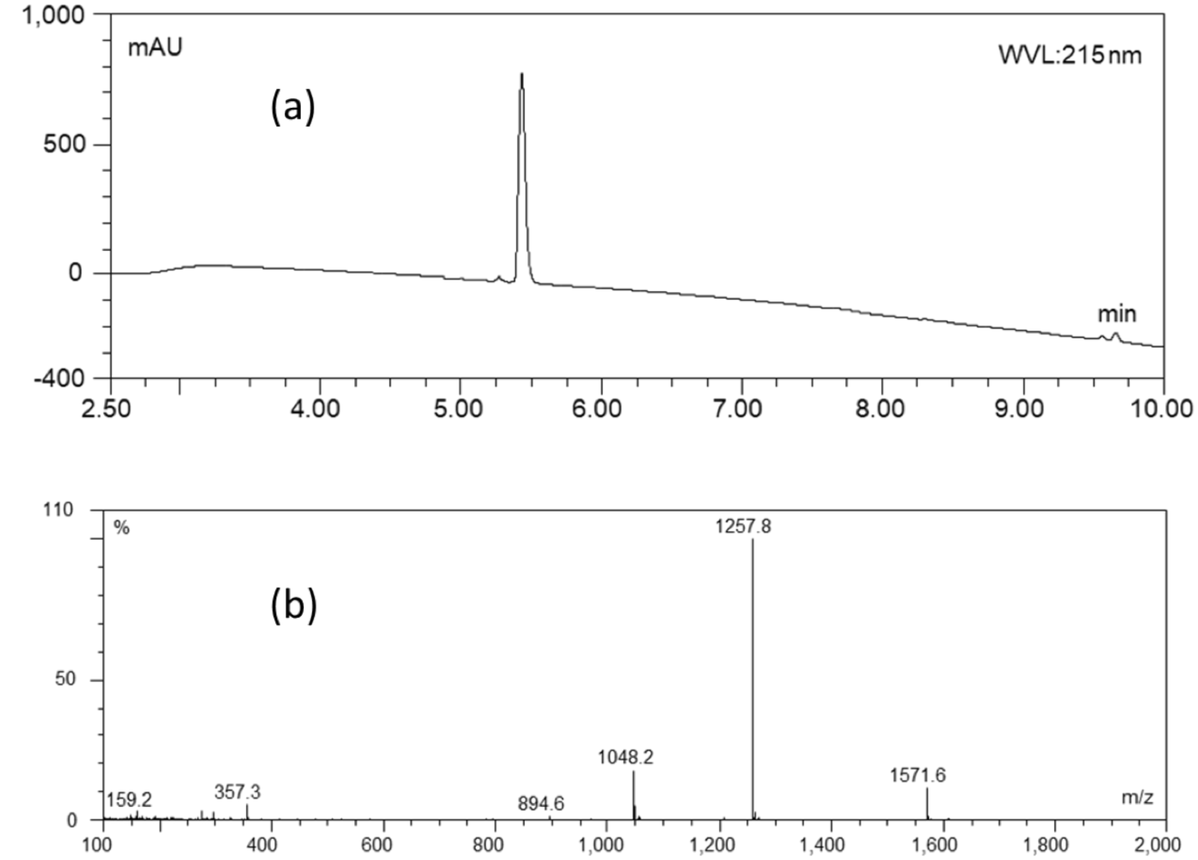

Supplementary Figure 6: After purification **a,** LCMS chromatogram of *Phe*^{B1}*Biotin-PEG$_3$*-HI **b,** Mass spectra of *Phe*^{B1}Biotin-PEG$_3$-HI

Serial No.	Peak	Calculated	Observed
1.	$[M]^+$	6250.78	Not Observed
2.	$[M+4H]^{4+}$	1563.45	1571.6
3.	$[M+5H]^{5+}$	1256.98	1257.8

Supplementary Table 4: LCMS Analysis of Phe^{B1}*Biotin-PEG$_3$*-HI

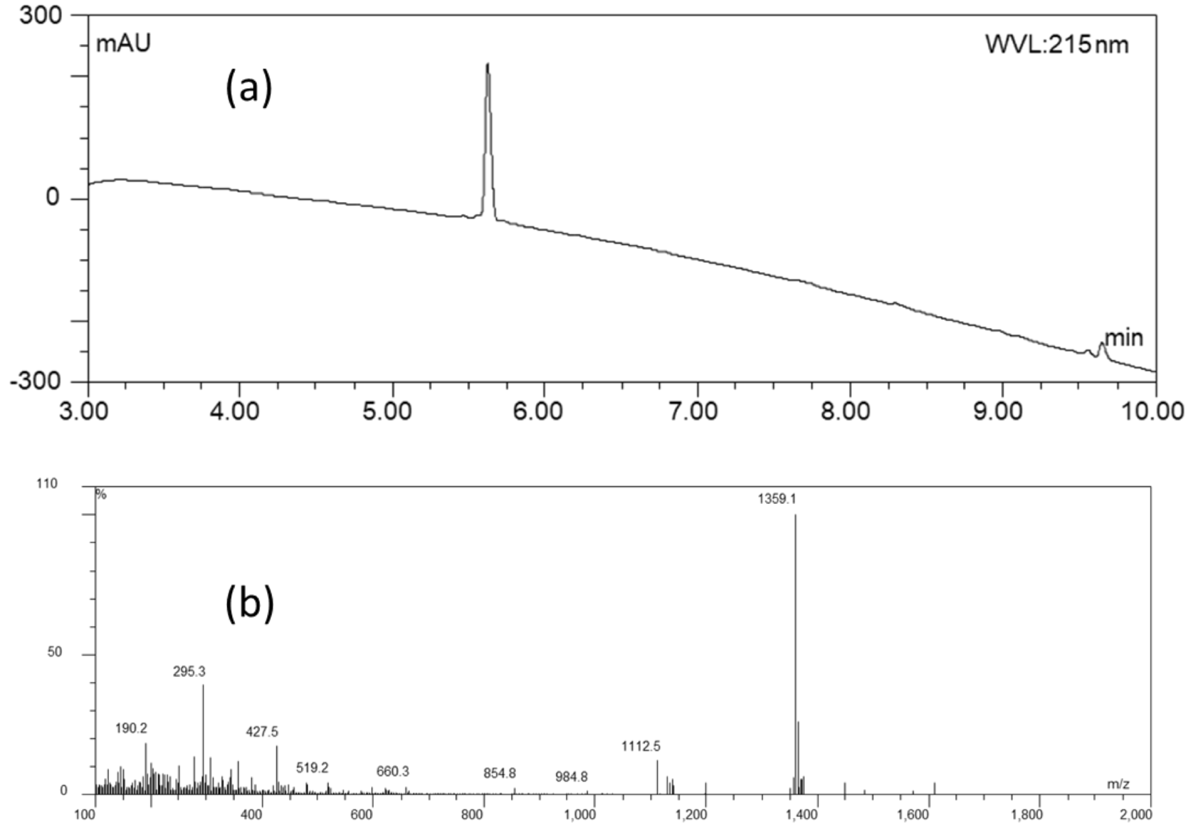

Supplementary Figure 7: After purification **a,** LCMS chromatogram of Phe^{B1}*Biotin-PEG$_3$*-Lys^{B29}*Atto-655*-HI **b,** Mass spectra of Phe^{B1}*Biotin-PEG$_3$*-Lys^{B29}*Atto-655*-HI

Serial No.	Peak	Calculated	Observed
1.	$[M]^{+}$	6787.063	Not Observed
2.	$[M+4H]^{4+}$	1358.820	1359.10

Supplementary Table 5: LCMS Analysis of Phe^{B1}-*Biotin-PEG$_3$*-Lys^{B29}*Atto-655*-HI

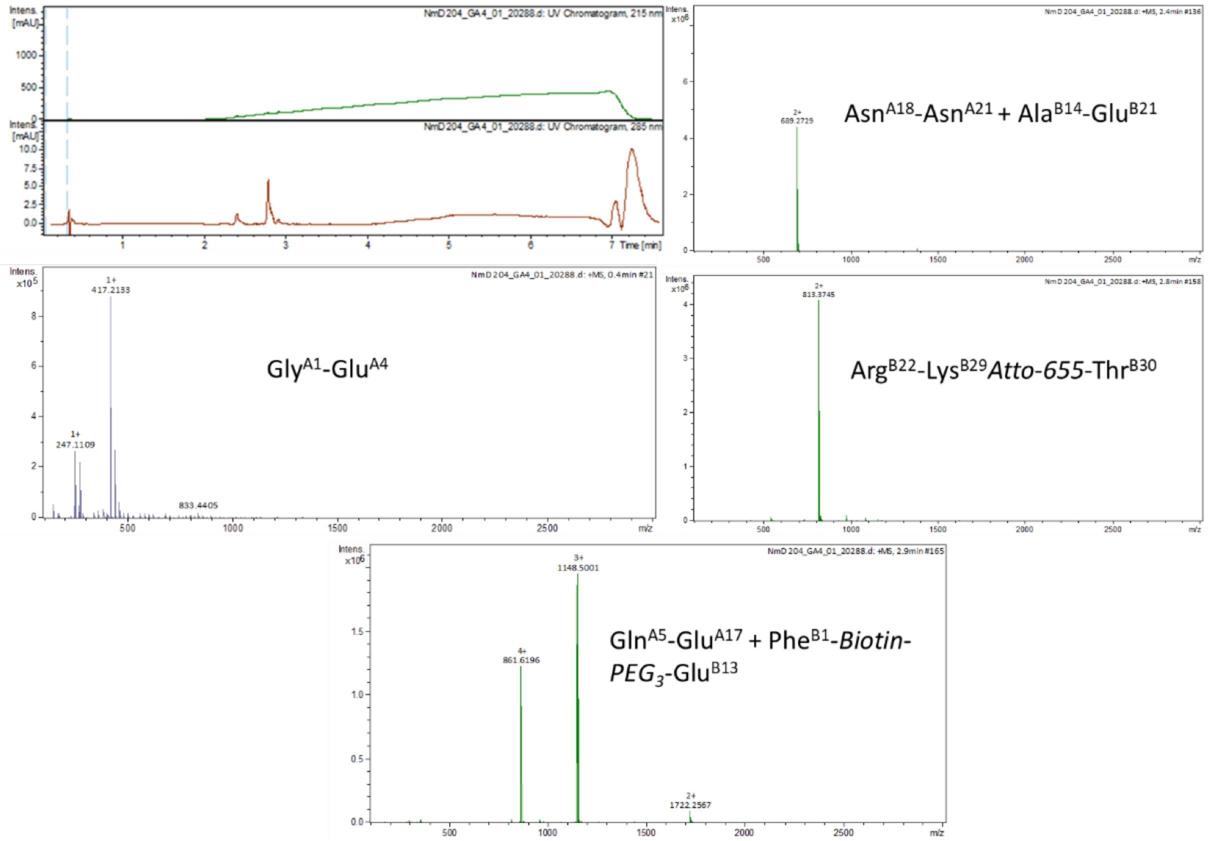

Supplementary Figure 8: LCMS chromatogram and mass spectra of Phe^B1^*Biotin-PEG*$_3$-Lys^B29^*Atto-655*-HI after V8 enzymatic cleavage.

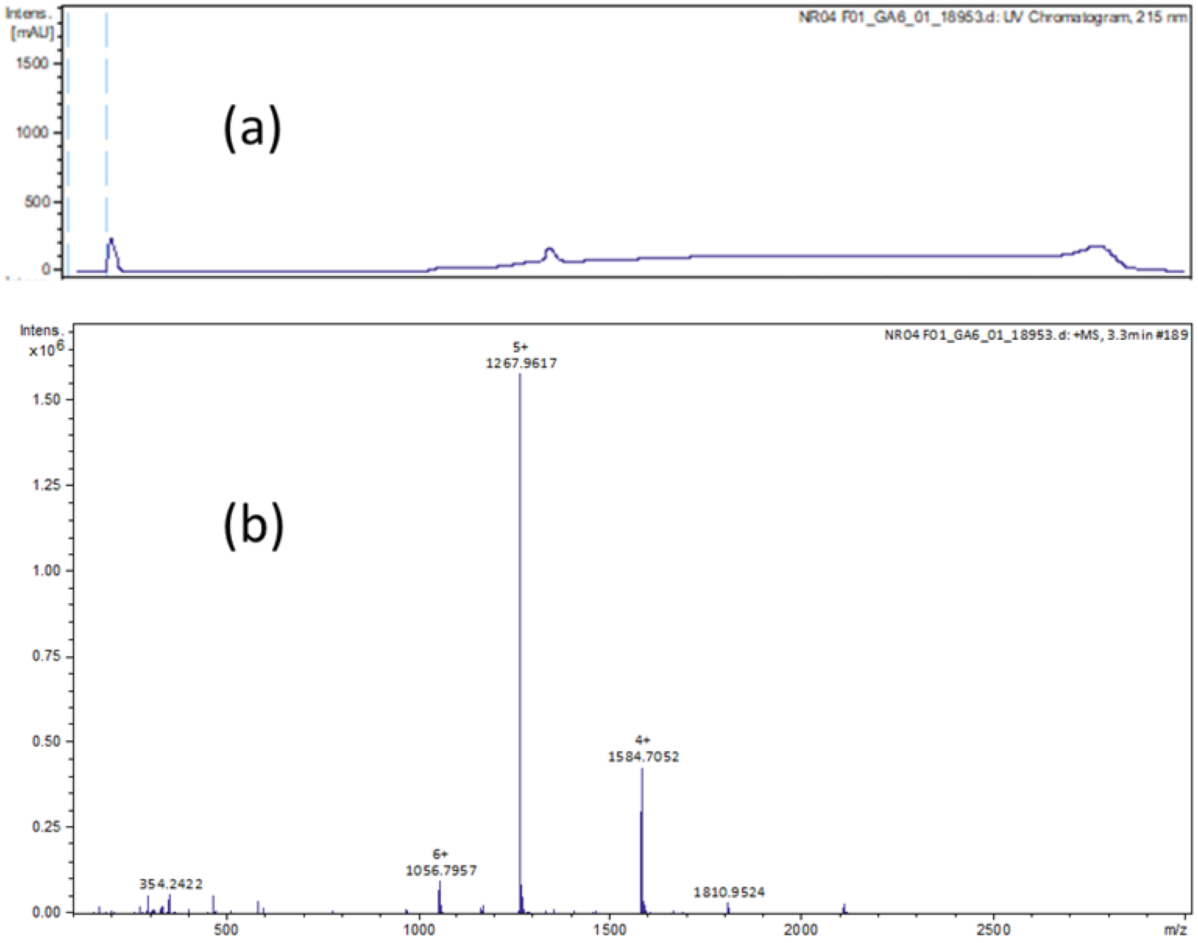

Supplementary Figure 9: After purification **a,** LCMS chromatogram of NovoRapid-Atto655 **b,** Mass spectra of LysB29-Atto655 NovoRapid HI.

Serial No.	Peak	Calculated	Observed
1.	$[M]^{+}$	6331.81	Not Observed
2.	$[M+4H]^{4+}$	1584.46	1584.70
3.	$[M+5H]^{5+}$	1267.77	1267.96
4.	$[M+6H]^{6+}$	1056.64	1056.79

Supplementary Table 6: LCMS Analysis of Phe^{B1}-*Biotin-PEG*$_3$-Lys^{B29}*Atto-655*-HI

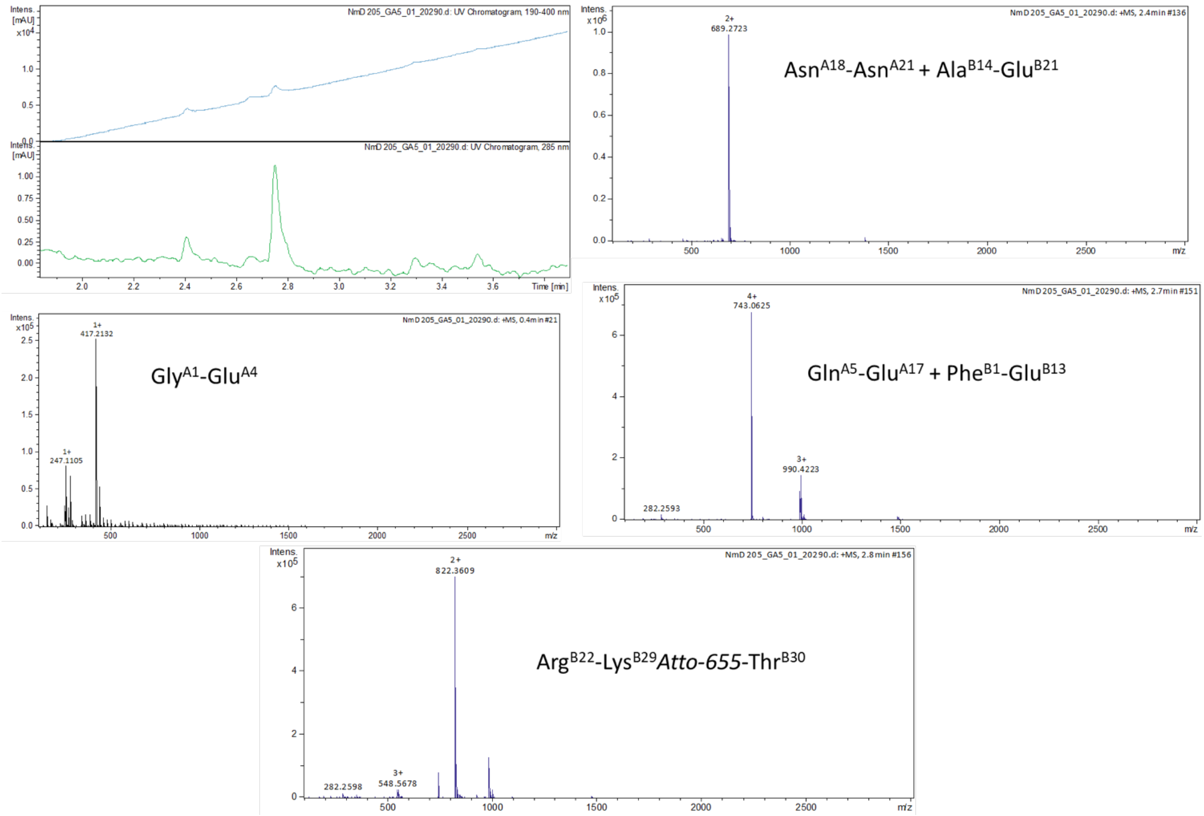

Supplementary Figure 10: LCMS chromatogram and mass spectra of Lys^{B29}-*Atto 655*-aspart insulin after V8 enzymatic cleavage

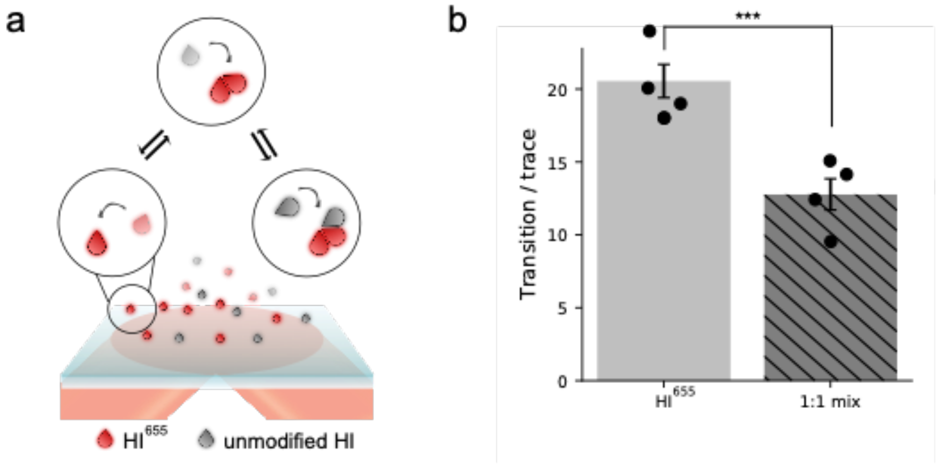

Supplementary Figure 11: 1:1 mixture of 10 nM HI^{655} and unmodified HI showed close to 50 % reduction of transitions per trace confirming that the label has little effect on the kinetics. **a,** Representation (not to scale) of the experimental setup. ATTO655 labeled Human Insulin (HI^{655}), are detected upon binding to PLL-PEG/biotin microscopy surface for hexamerization. Particles in solution are not detected due to decay of the evanescence field excitation (pink/shaded red). Unmodified insulin (grey) is not detected either. **b,** Transition pr trace for the two conditions.

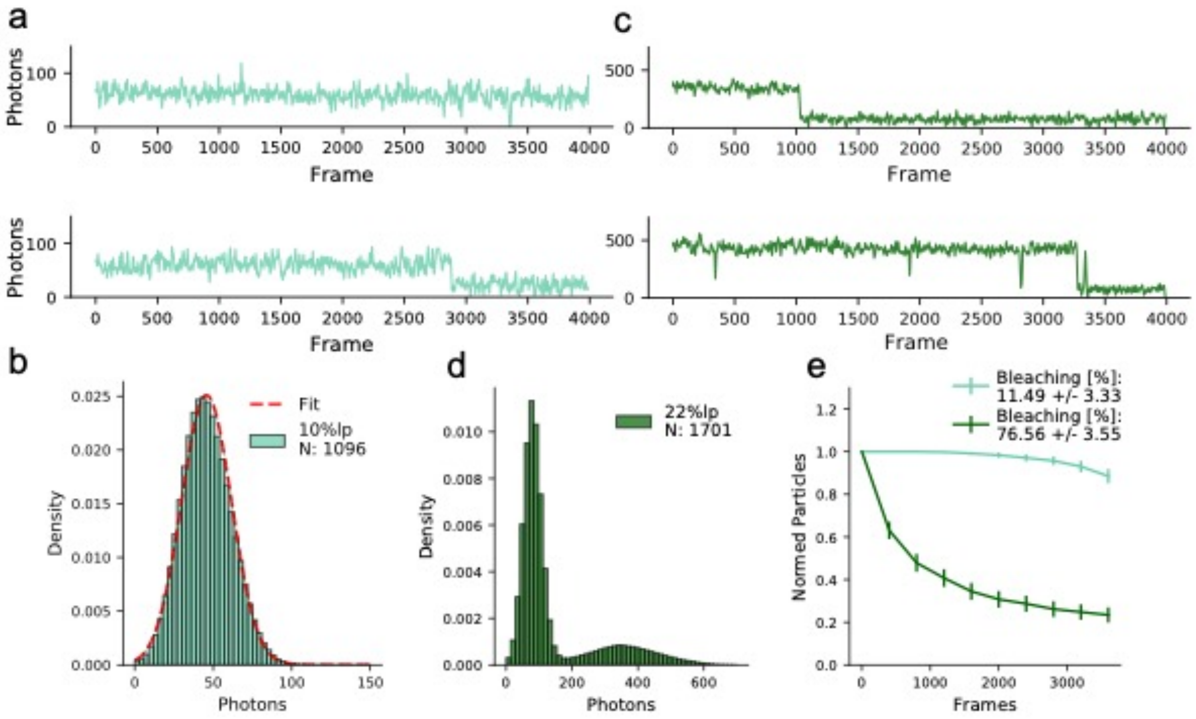

Supplementary Figure 12: HI^{655}-Biotin control experiment showed fluorophore blinking and bleaching as negligible since we only observe 0.6 % blinking and 12 % bleaching, ensuring minimum bias on observed fluorescent readout. **a,** Representative traces of single ATTO655 fluorophores coupled to HI. Top shows no bleaching, while bottom shows a single bleaching step for representation. Laser power = 10 % **b,** Intensity profile for ATTO655 at 10 % laser power. Fit with a Gaussian distribution (red dotted line) reveals a mean of 46 ± 16 photons (n_{videos} = 12). **c,** Representative traces of single ATTO655 fluorophores at 22 % laser power. Both display single bleaching events, indicative of fluorescent monolabelling. **d,** Intensity profile for ATTO655 at 22 % laser power showing two distinct distributions. One represents the background, while the other represents ATTO655. **e,** Quantification of bleaching for 10 % and 22 % laser power reveals 12 % and close to 80 % bleaching respectively, in the full experimental timeframe of 4000 frames. Error bars are the standard deviation from 12 movies respectively.

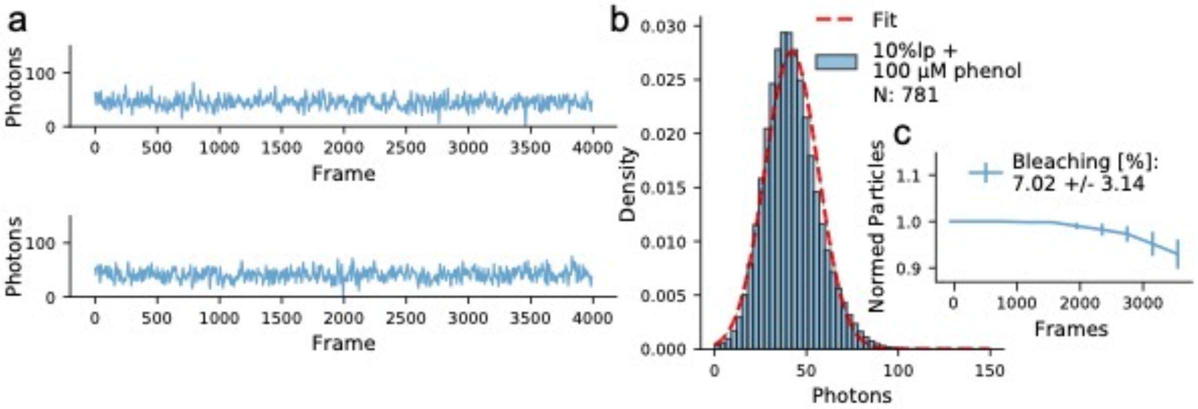

Supplementary Figure 13: HI^{655}-Biotin control experiment with 100 mikroM phenol showed fluorophore blinking and bleaching as negligible since we only observe 0.5 % blinking and 7 % bleaching, ensuring minimum bias on observed fluorescent readout. **a,** Representative traces of single ATTO655 fluorophores coupled to HI. Laser power = 10 % **b,** Intensity profile for ATTO655 at 10 % laser power. Fit with a Gaussian distribution (red dotted line) reveals a mean of 41 ± 14 photons (N_{videos} = 12) **c,** Quantification of bleaching for 10 % laser power reveals 7 % bleaching, in the full experimental timeframe of 4000 frames. Error bars are the standard deviation from 12 movies respectively.

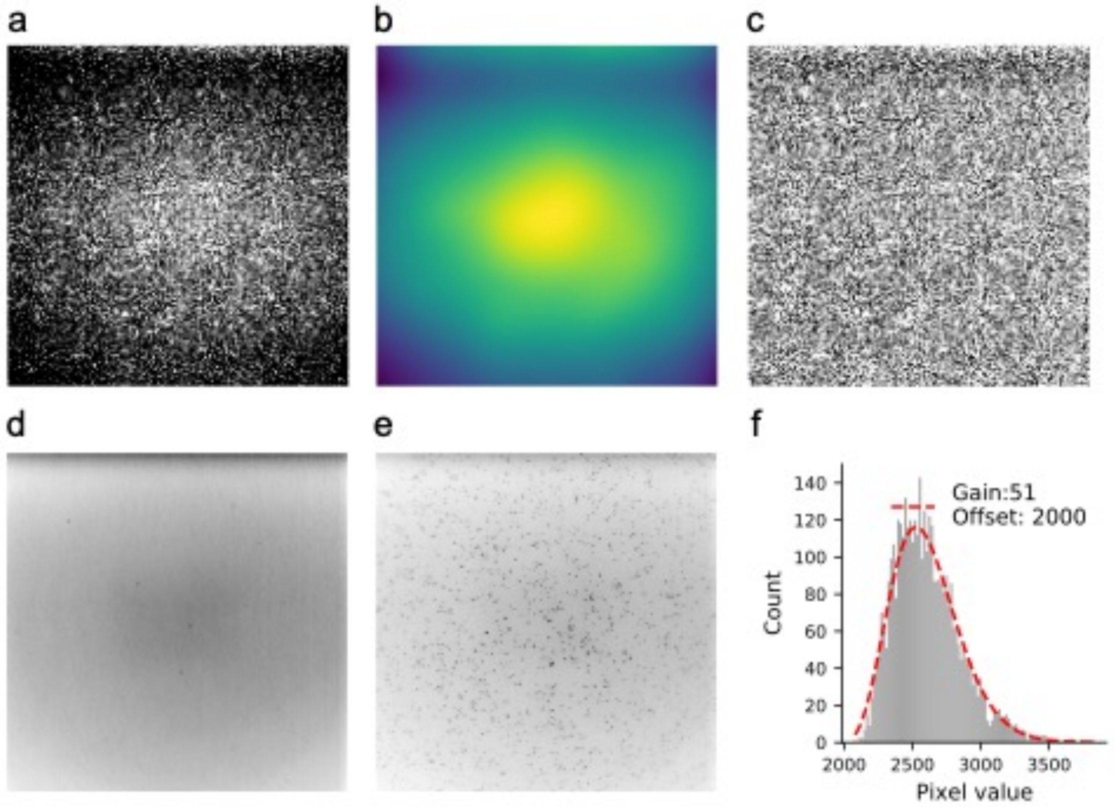

Supplementary Figure 14: a-c: Illumination profile correction. **a,** 512 x 512 pixel microscope image with nonspecifically bound fluorophores before correcting for uneven illumination profile. **b,** Illumination profile estimated. **c,** The same microscope image after correcting for uneven illumination profile. d-f: Electron multiplying charge coupled device (EMCCD) calibration directly from a background movie. **d,** Time averaged movie of a background movie before adding fluorescent particles. **e,** Time averaged movie of a movie after adding fluorescent particles. **f,** EMCCD fit of a random pixel in d, resulting in Gain and Offset.

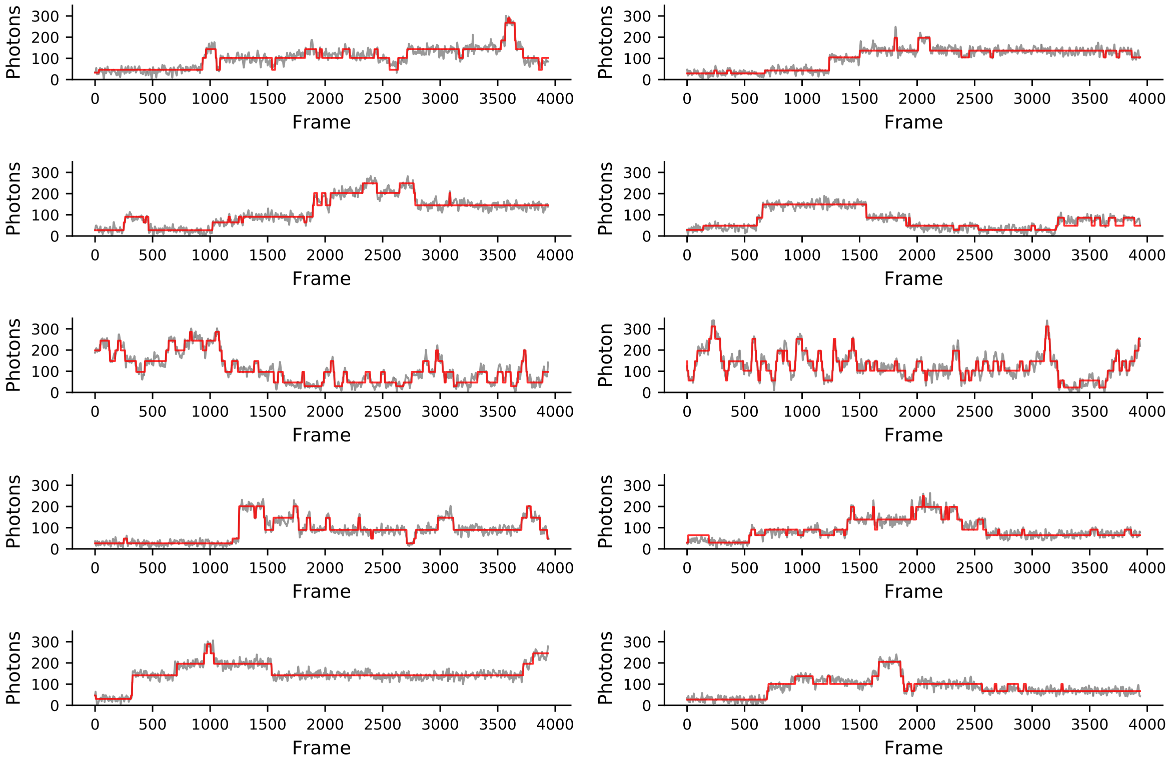

Supplementary Figure 15: Representative trajectories overlayed with idealized trace from HMM prediction.

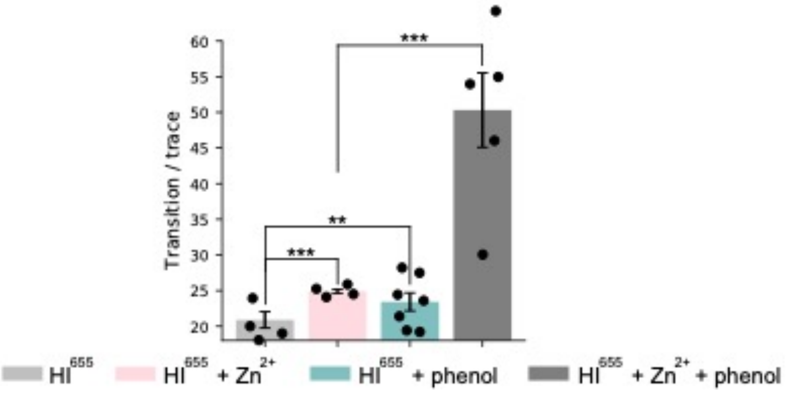

Supplementary Figure 16: Transition per trace show increased dynamics for 10 nM HI^{655} (20.90 ±1.14) upon addition of Zn^{2+} (p-value = 0.0004 (24.90 ± 0.26)) and upon addition with phenol (p-value = 0.009 (23.42 ± 1.26)). Transitions pr trace increase even more when both Zn^{2+} and phenol is added together (p-value = 1.2 * 10^{-5} (50.32 ± 5.23)). (Level of significance is determined by a Welch's t-test (*p-value < 0.05; **p-value < 0.01; ***p-value < 0.001).

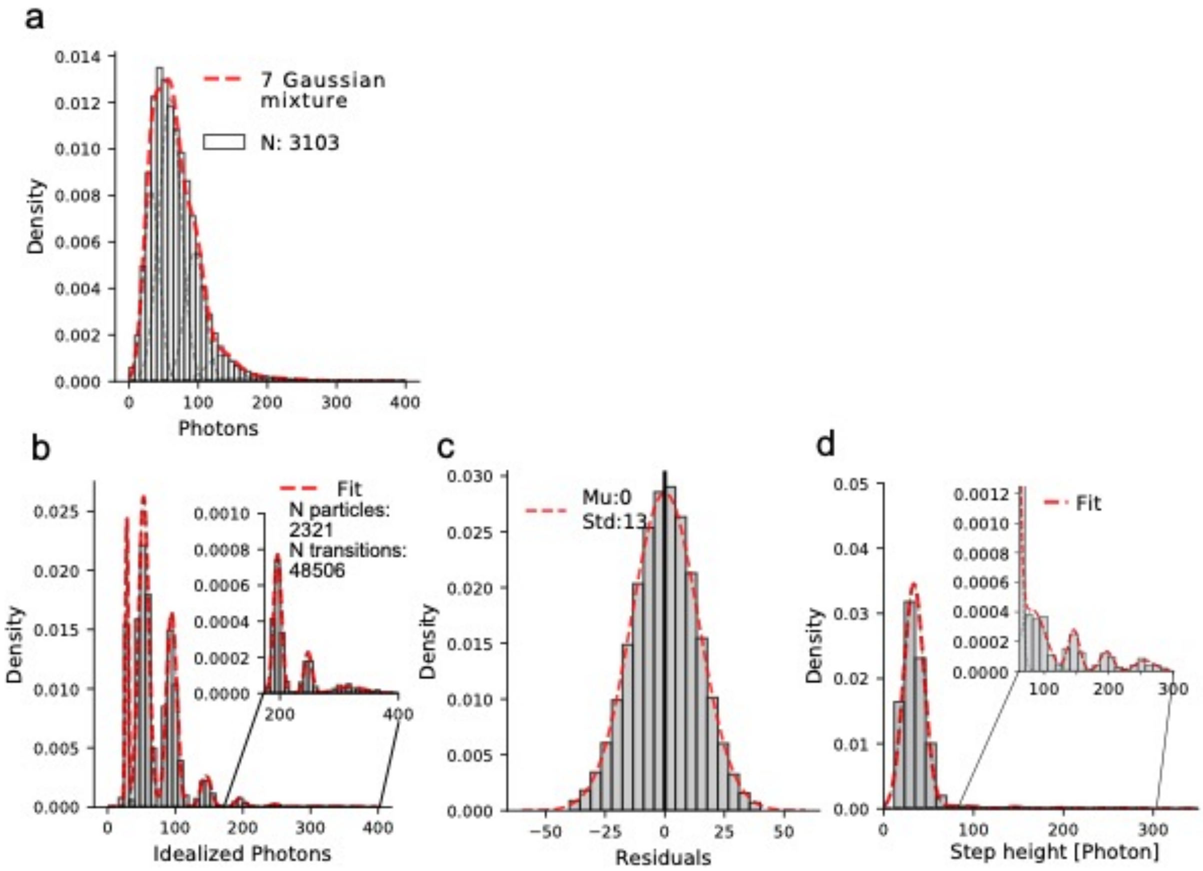

Supplementary Figure 17: Hidden Markov Modeling of HI^{655} **a,** Traces for HI^{655} grouped in a histogram. Red line shows fit with a Gaussian mixture model of seven distinct Gaussian distributions indicating that a seven-state model indeed reflects the photon counts observed. N = 3013 is the total number of trajectories before HMM analysis. **b,** Idealized photon histogram for 10 nM HI^{655} with a Gaussian mixture model fit to the seven distinct distributions. **c,** Residuals for experiments with10 nM HI^{655} grouped in histograms and fitted with Gaussian. μ and σ are displayed for each condition and show no systematic error of HMM fit. **d,** Estimation of oligomer concentration in solution for 10 nM HI^{655}. Histograms show stepheight (essentially the difference between photon count after a transition and photon count before a transition). Distributions correspond to monomer addition, dimer addition, trimer addition, tetramer addition and pentamer addition. Histograms are fitted with five Gaussian distributions to get weight for each distribution as an estimation of oligomer concentration in solution.

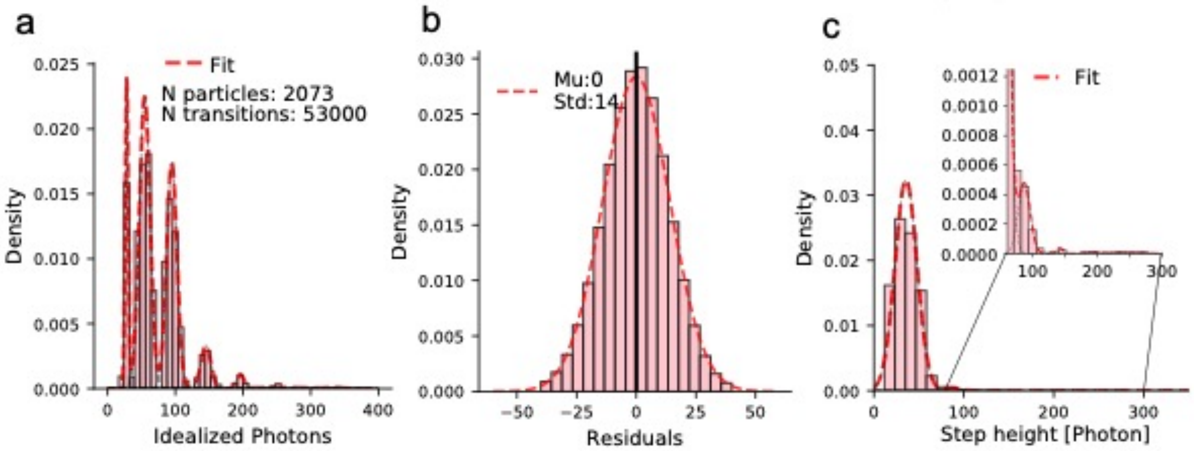

Supplementary Figure 18: Hidden Markov Modeling of 10 nM HI^{655} under addition of Zn^{2+} **a,** Idealized photon histogram for 10 nM + 100 µM Zn^{2+} with a Gaussian mixture model fit to the seven distinct distributions. **b,** Residuals for experiments with 10 nM HI^{655} + 100 µM Zn^{2+} grouped in histogram and fitted with Gaussian. μ and σ are displayed and show no systematic error in HMM fit. **c,** Estimation of oligomer concentration on solution. Histogram of stepheight (essentially difference between photon count after a transition and photon count before a transition). Distributions correspond to monomer addition, dimer addition, trimer addition, tetramer addition and pentamer addition. Histogram fitted with five Gaussian distributions to get weight for each distribution.

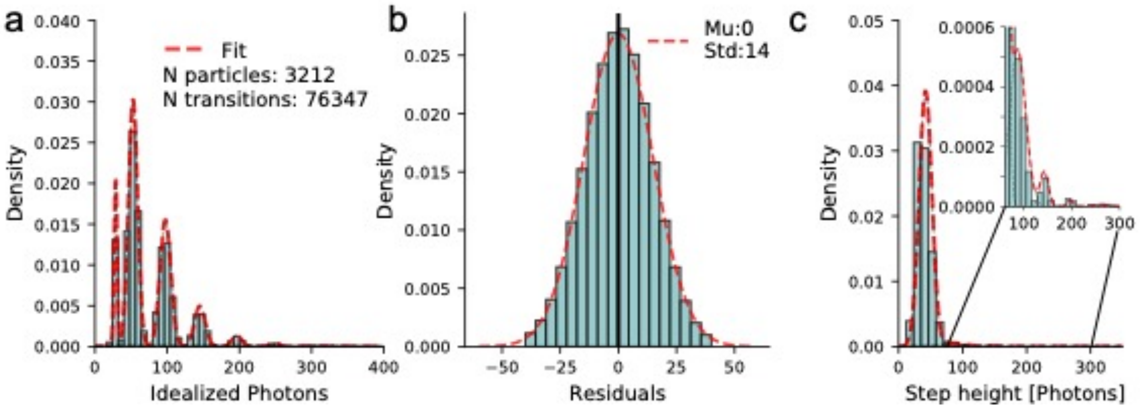

Supplementary Figure 19: Hidden Markov Modeling of 10 nM HI[655] under addition of phenol **a,** Idealized photon histogram for 10 nM + 25 µM phenol with a Gaussian mixture model fit to the seven distinct distributions. **b,** Residuals for experiments with 10 nM HI[655] + 25 µM phenol grouped in histogram and fitted with Gaussian. µ and σ are displayed and show no systematic error in HMM fit. **c,** Estimation of oligomer concentration on solution. Histogram of stepheight (essentially difference between photon count after a transition and photon count before a transition). Distributions correspond to monomer addition, dimer addition, trimer addition, tetramer addition and pentamer addition. Histogram fitted with five Gaussian distributions to get weight for each distribution.

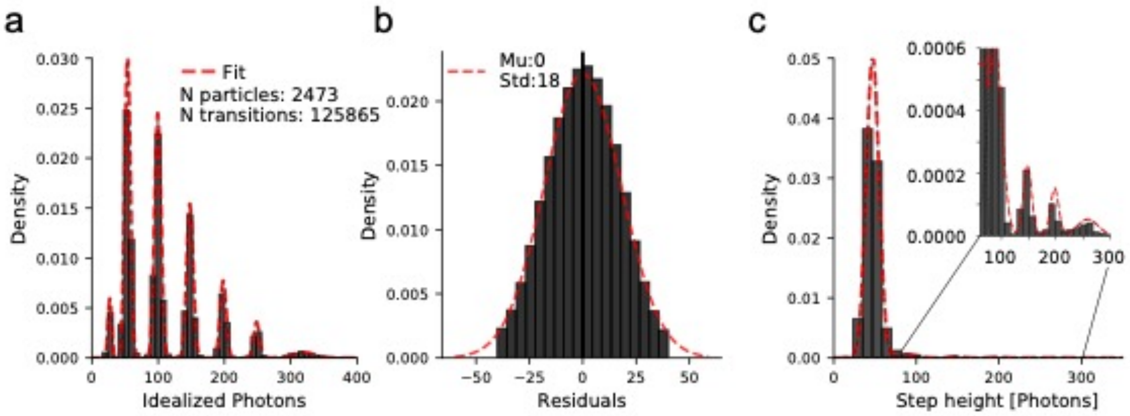

Supplementary Figure 20: Hidden Markov Modeling of 10 nM HI^{655} under addition of Zn^{2+} and phenol **a,** Idealized photon histogram for 10 nM + 100 µM Zn^{2+} + 25 µM phenol with a Gaussian mixture model fit to the seven distinct distributions. **b,** Residuals for experiments with 10 nM HI^{655} + 100 µM Zn^{2+} + 25 µM phenol grouped in histogram and fitted with Gaussian. µ and σ are displayed and show no systematic error in HMM fit. **c,** Estimation of oligomer concentration on solution. Histogram of stepheight (essentially difference between photon count after a transition and photon count before a transition). Distributions correspond to monomer addition, dimer addition, trimer addition, tetramer addition and pentamer addition. Histogram fitted with five Gaussian distributions to get weight for each distribution.

	Monomer	Dimer	Trimer	Tetramer	Pentamer	Hexamer
10 nM HI^{655}	56.6 ± 0.0 %	35.5 ± 0.0 %	5.8 ± 0.0 %	1.5 ± 0.0 %	0.4 ± 0.0 %	0.2 ± 0.0 %
10 nM HI^{655} + 100 µM Zn^{2+}	51.7 ± 0.0 %	38.6 ± 0.0 %	6.9 ± 0.0 %	1.7 ± 0.0 %	0.8 ± 0.1 %	0.2 ± 0.1 %
10 nM $HI^{655}I$ + 25 µM phenol	53.6 ± 0.0 %	32.5 ± 0.0 %	10.2 ± 0.0 %	2.5 ± 0.0 %	0.7 ± 0.0 %	0.4 ± 0.0 %
10 nM $HI^{655}I$ + Zn^{2+} + phenol	33.8 ± 0.0 %	30.9 ± 0.0 %	19.4 ± 0.0 %	9.2 ± 0.0 %	4.2 ± 0.0 %	2.5 ± 0.0 %

Supplementary Table 7: State occupancies from Gaussian fit for 10 nM HI^{655} with addition of Zn^{2+} and phenol. Error bars are SEM from 10 x bootstrap fit to the idealized histogram.

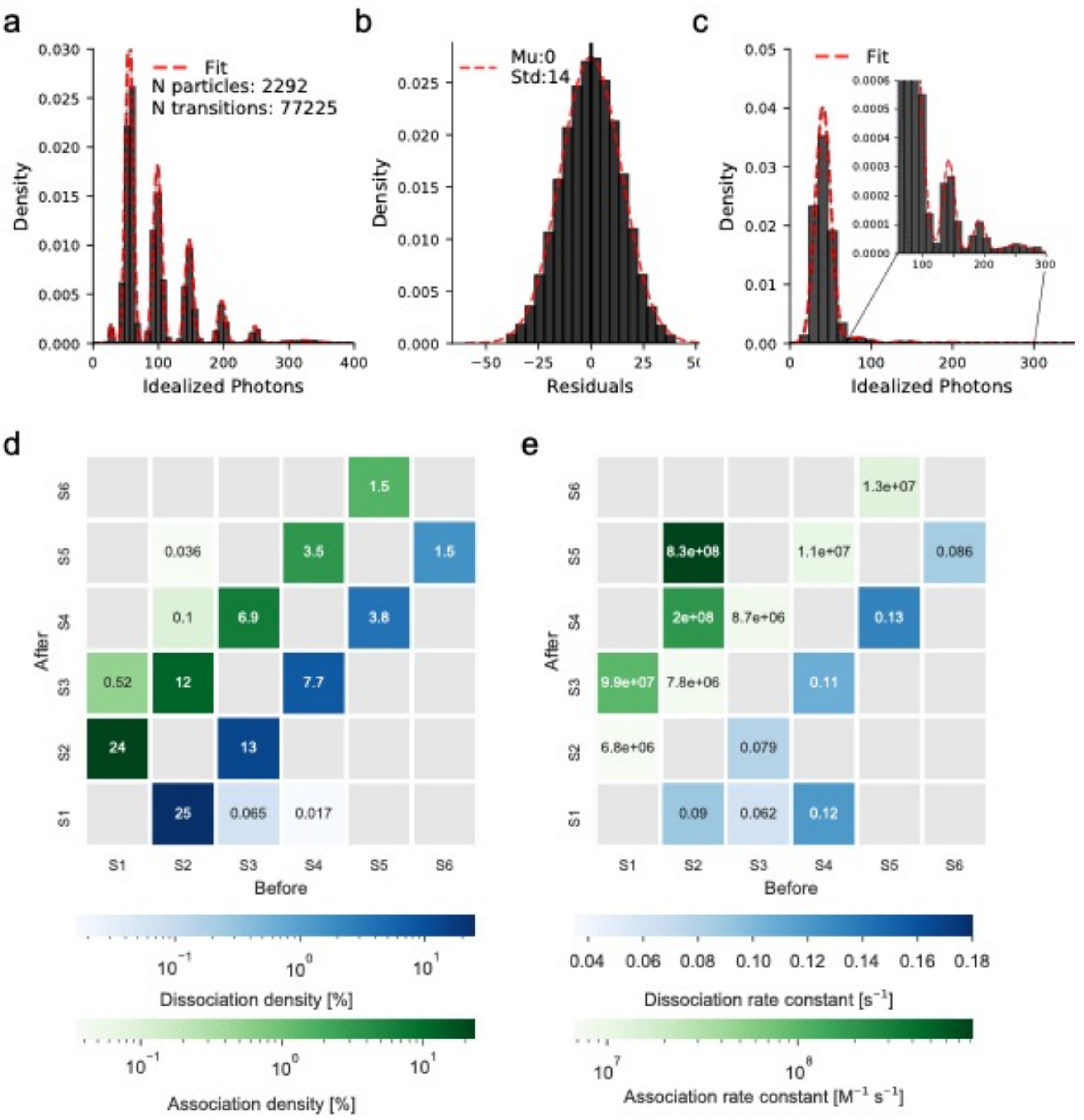

Supplementary Figure 21: Hidden Markov Modeling of 10 nM NovoRapid[655]. **a,** Idealized photon histogram with a Gaussian mixture model fit to the seven distinct distributions. **b,** Residuals for experiments grouped in histogram and fitted with Gaussian. μ and σ are displayed and show no systematic error in HMM fit. **c,** Estimation of oligomer concentration on solution. Histogram of stepheight (essentially difference between photon count after a transition and photon count before a transition). Distributions correspond to monomer addition, dimer addition, trimer addition, tetramer addition and pentamer addition. Histogram fitted with five Gaussian distributions to get weight for each distribution. **d-e,** CHESS (Complete HEatmap of State transitionS) plot shows thermodynamic (d) and kinetic (e) parameters for each separate transition from a before state (x-axis) to an after state (y-axis). The numbers within the squares are the value for transition density. Gray squares are transitions with no data points. Transitions involving S0 (background) have been removed. Triangle denotes if less than 10 transitions were observed.

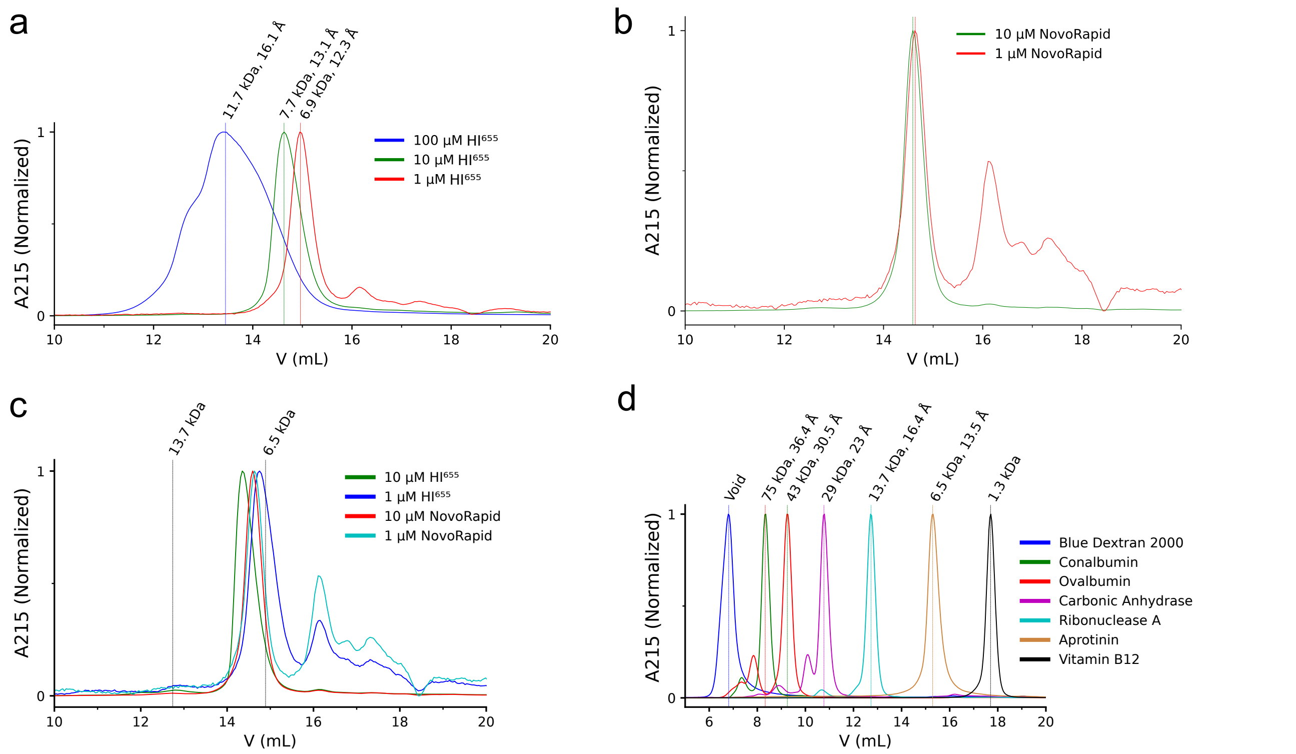

Supplementary Figure 22: Size exclusion chromatography (SEC) experiments at varying concentration. **a,** SEC of HI^{655} at 100 µM, 10 µM, and 1 µM. The elution peak of 100 µM HI^{655} at 13.45 mL was 11.7 kDa with Rs of 16.1 Å. The elution peak of 10 µM HI^{655} at 14.63 mL was 7.7 kDa with Rs of 13.1 Å. The elution peak of 1 µM HI^{655} at 14.96 mL was 6.9 kDa with Rs of 12.3 Å. **b,** SEC of Atto655 modified NovoRapid at 10 µM and 1 µM. The elution peak of 10 µM Atto655-NovoRapid at 14.59 mL was 7.9 kDa with *Rs* of 13.2 Å. The elution peak of 1 µM Atto655-NovoRapid at 14.64 mL was 7.7 kDa with *Rs* of 13.1 Å. **c,** SEC comparisons between 10 µM and 1 µM HI^{655} and Atto655-NovoRapid. Dotted lines depict the MW of standard proteins. The average size of HI^{655} is increased by increasing concentration consistent with an equilibrium shift to a higher oligomeric state. NovoRapid's equilibrium remains unaltered on the other hand and appears to remain in a monomeric state. **d,** SEC of standard calibration proteins, including Blue Dextran 2000 and Vitamin B12, carried on a Superdex 75 Increase 10/300 GL column. The MW and R_s of proteins are shown at their respective peak top.

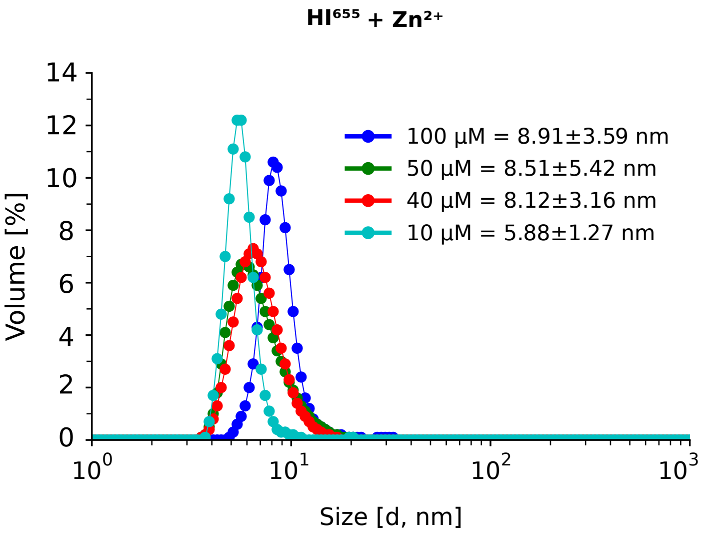

Supplementary Figure 23: Dynamic Light Scattering experiments measurements at 100, 50, 40 and 10 µM HI^{655} + Zn^{2+} in 10 mM Na_2HPO_4, 10 mM Na_2HPO_4, 10 mM NaH_2PO4, and 5 mM NaCl at pH 7.5. Increasing the insulin concentration result as expected to shift in hydrodynamic radius consistent with shift in equilibrium towards higher order oligomers.

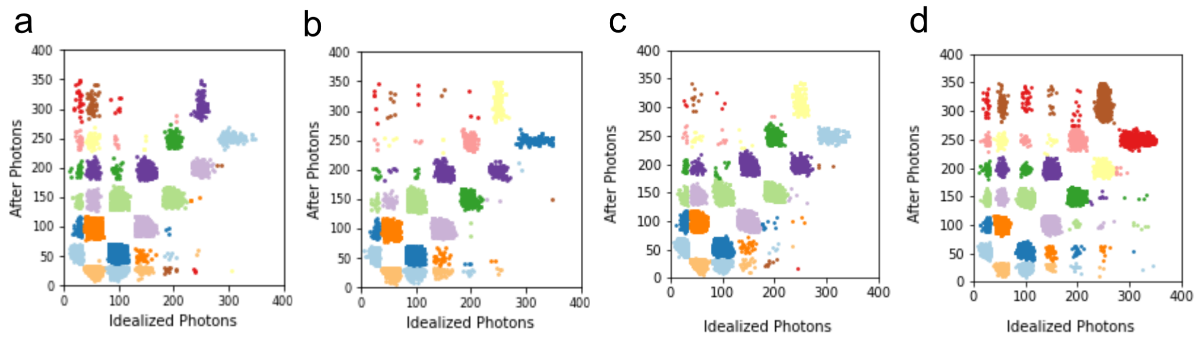

Supplementary Figure 24: Transition scatter plot of photon count before a transition and after a transition for experiments with **a,** 10 nM HI^{655}, **b,** 10 nM HI^{655} + 100 μM Zn^{2+}, **c,** 10 nM HI^{655} + 25 μM phenol and **d,** 10 nM HI^{655} + 100 μM Zn^{2+} + 25 μM phenol. The color of each cluster has no significance, other than visually easy separation.

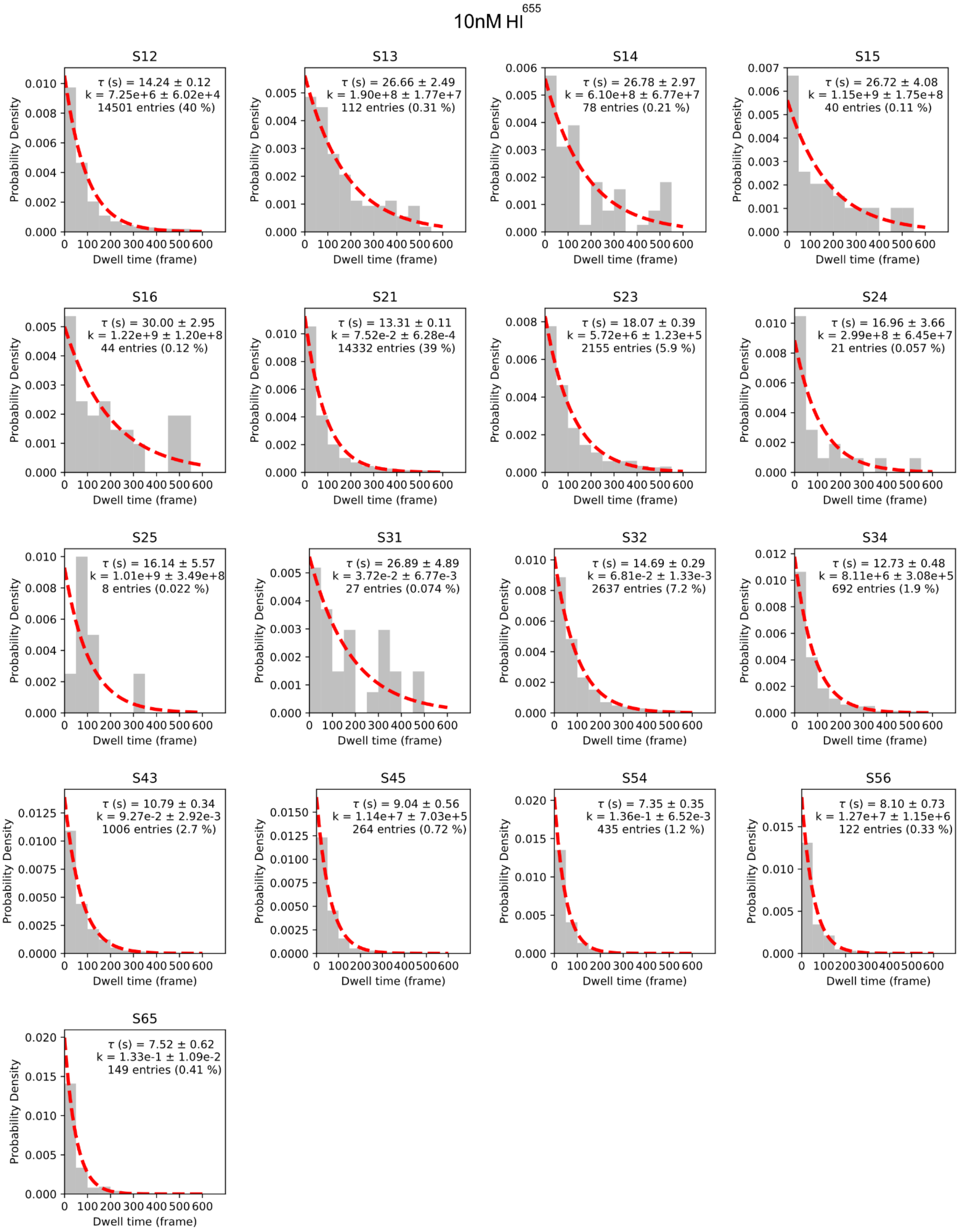

Supplementary Figure 25: Single exponential fit (red dotted line) to dwell times (gray) for 17 separable clusters of unique transitions for 10 nM HI^{655}. Within each fit is the extracted overall dwell time, rate constant and density (how many times the transition is observed) shown.

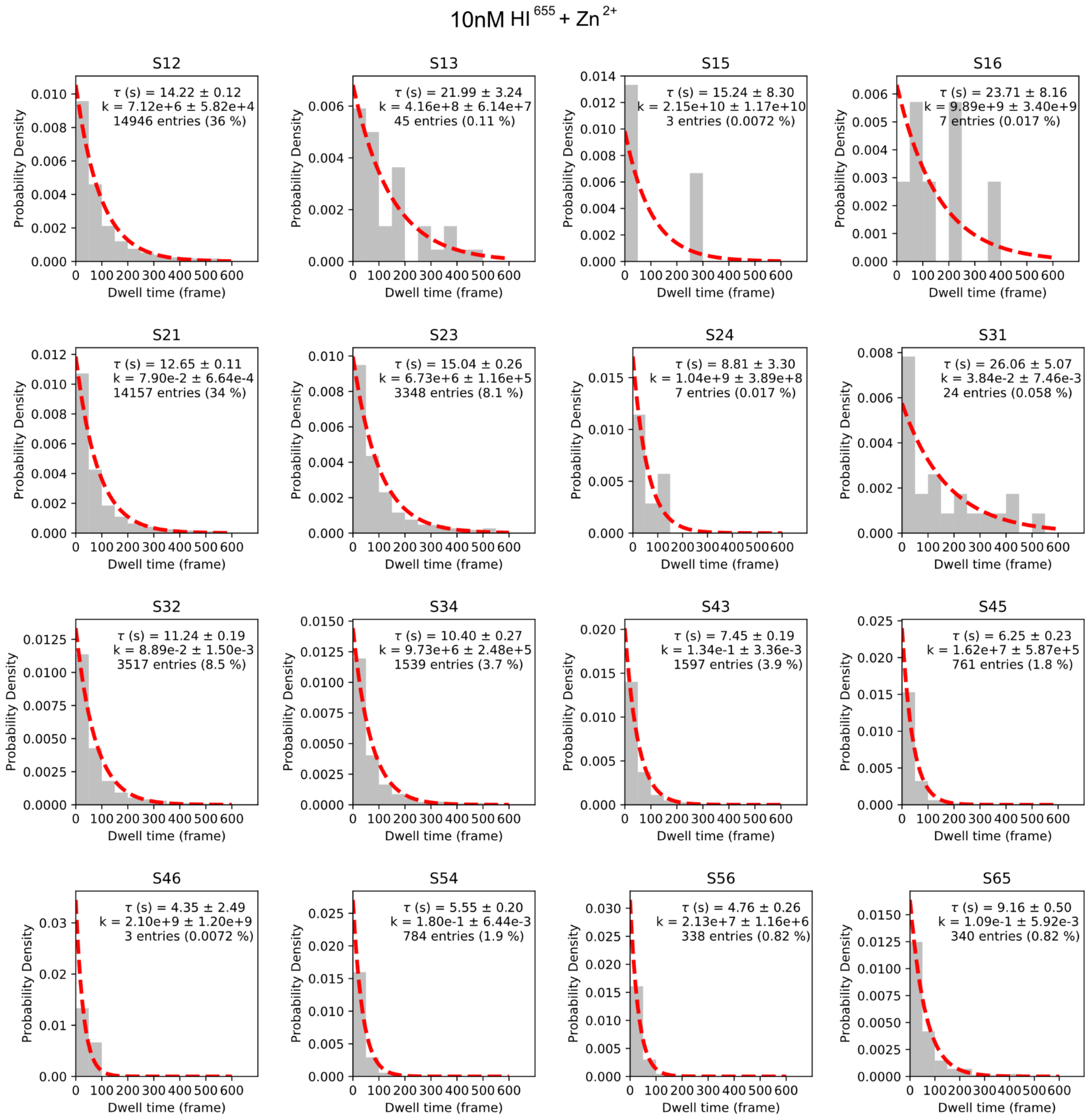

Supplementary Figure 26: Single exponential fit (red dotted line) to dwell times (gray) for 17 separable clusters of unique transitions for 10 nM HI^{655} + 100 µM Zn^{2+}. Within each fit is the extracted overall dwell time, rate constant and density (how many times the transition is observed) shown.

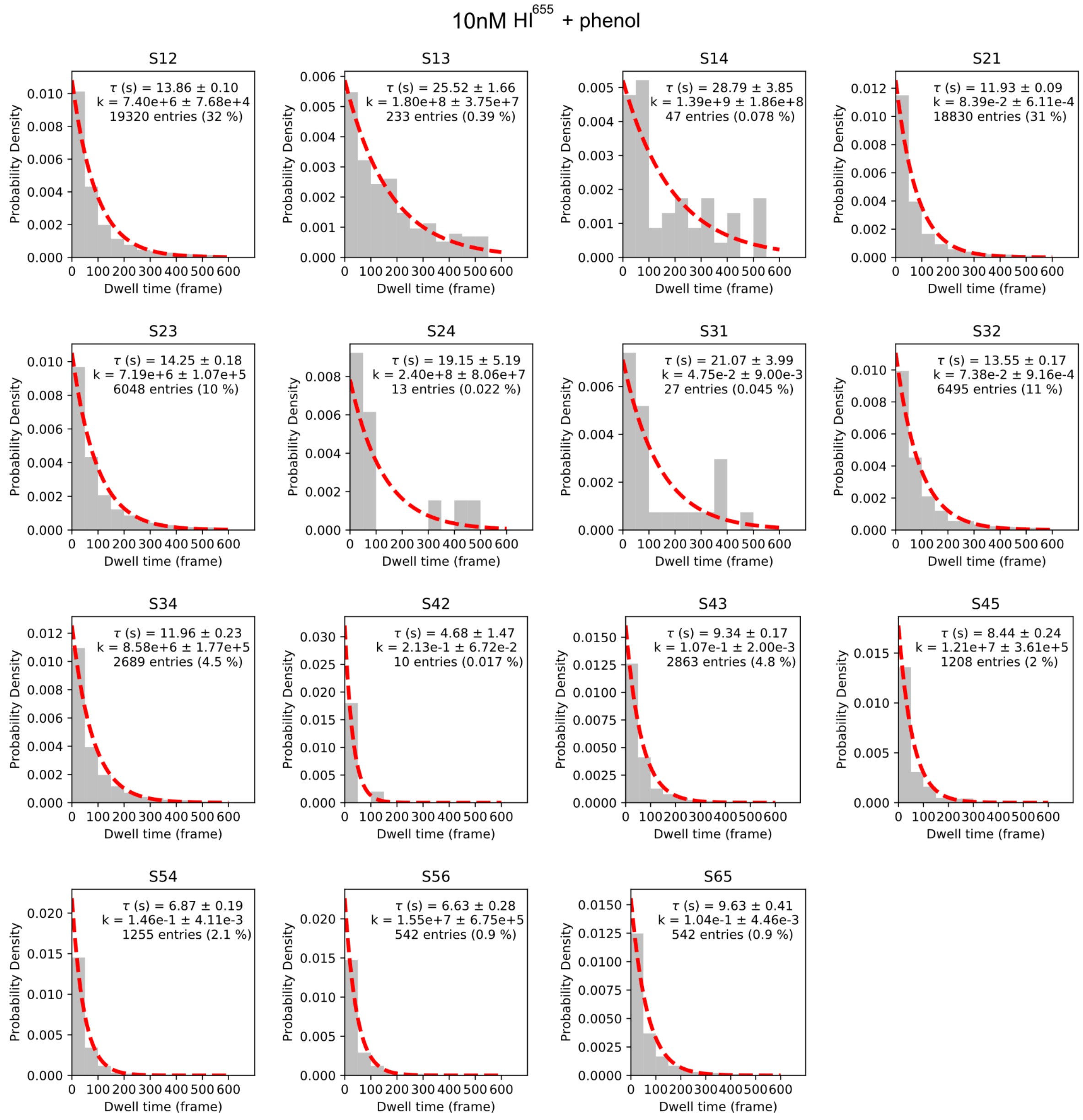

Supplementary Figure 27: Single exponential fit (red dotted line) to dwell times (gray) for 17 separable clusters of unique transitions for 10 nM HI^{655} + 25 μM phenol. Within each fit is the extracted overall dwell time, rate constant and density (how many times the transition is observed) shown.

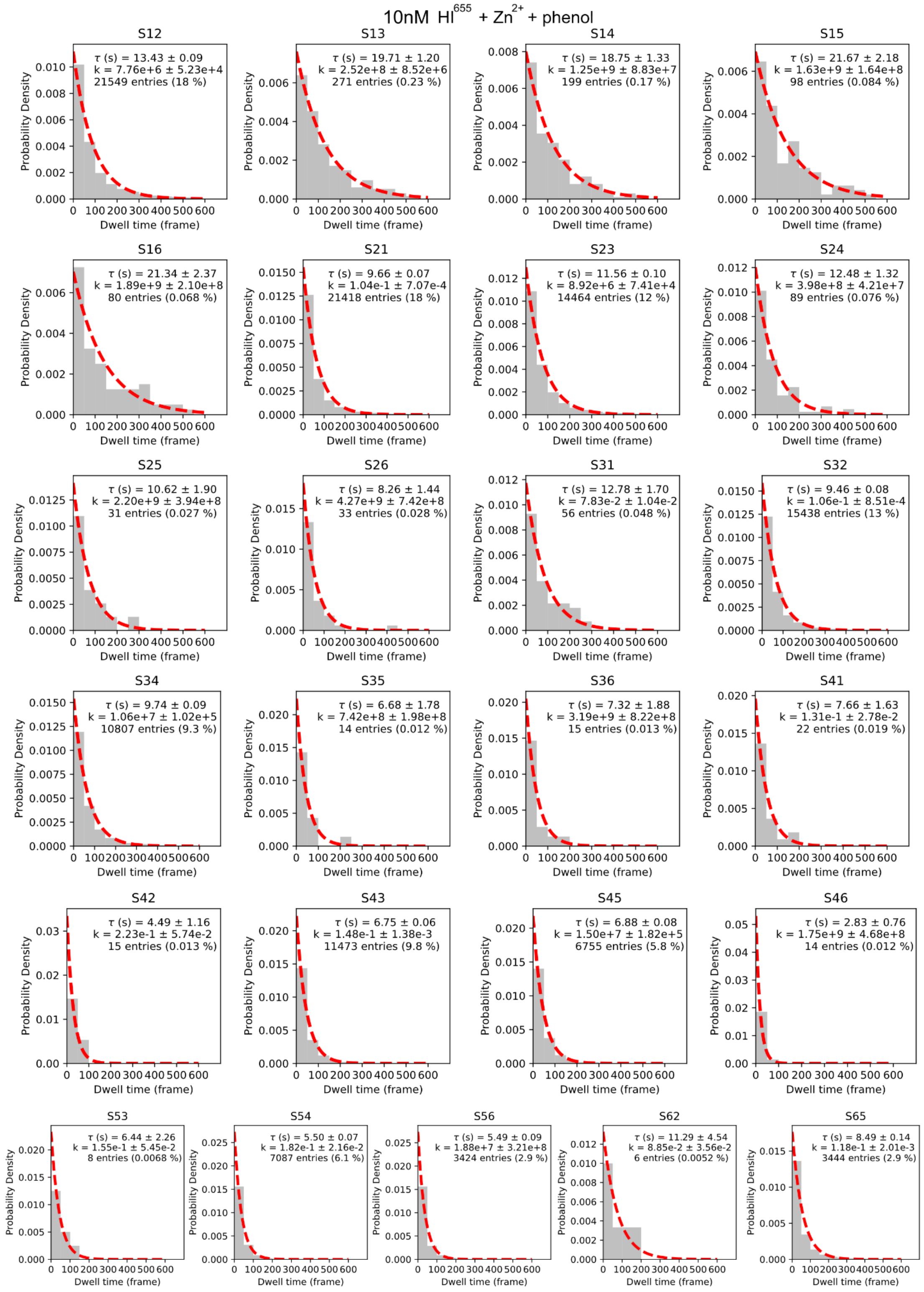

Supplementary Figure 28: Single exponential fit (red dotted line) to dwell times (gray) for 17 separable clusters of unique transitions for 10 nM HI^{655} + 100 µM Zn^{2+} + 25 µM phenol. Within each fit is the extracted overall dwell time, rate constant and density (how many times the transition is observed) shown.

S6	44	6	0	2	122	
S5	40	8	5	264		149
S4	78	21	692		435	2
S3	112	2155		1006	4	0
S2	14501		2637	3	0	0
S1		14332	27	6	2	0
	S1	S2	S3	S4	S5	S6

Supplementary Table 8: Raw count of transitions frequency for all observed transitions for 10 nM HI^{655}. Not all transitions have been observed. For some transitions with too little statistics, the rate could not be reliably extracted. Transitions involving S0 have been removed. N = 36653 transitions.

S6	9	2	0	2	542	
S5	13	6	5	1208		542
S4	47	13	2689		1255	3
S3	233	6048		2863	5	0
S2	19320		6495	10	3	0
S1		18830	27	5	1	0
	S1	S2	S3	S4	S5	S6

Supplementary Table 9: Raw count of transitions frequency for all observed transitions for 10 nM HI^{655} + 25 µM phenol . Not all transitions have been observed. For some transitions with too little statistics, the rate could not be reliably extracted. Transitions involving S0 have been removed. N = 60176 transitions.

S6	7	3	2	3	338	
S5	3	3	3	761		340
S4	5	7	1539		784	2
S3	45	3348		1597	2	1
S2	14946		3517	1	0	0
S1		14157	24	3	2	0
	S1	S2	S3	S4	S5	S6

Supplementary Table 10: Raw count of transitions frequency for all observed transitions for 10 nM HI^{655} + 100 µM Zn^{2+}. Not all transitions have been observed. For some transitions with too little statistics, the rate could not be reliably extracted. Transitions involving S0 have been removed. N = 41443 transitions.

S6	80	33	15	14	3424	
S5	98	31	14	6755		3444
S4	199	89	10807		7087	7
S3	271	14464		11473	8	3
S2	21549		15438	15	8	6
S1		21418	56	22	11	1
	S1	S2	S3	S4	S5	S6

Supplementary Table 11: Raw count of transitions frequency for all observed transitions for 10 nM HI^{655} + 100 µM Zn^{2+} + 25 µM phenol. Not all transitions have been observed. For some transitions with too little statistics, the rate could not be reliably extracted. Transitions involving S0 have been removed. N = 116840 transitions.

10 nM HI[655]			
Transition	$k_{association}$ [s^{-1} M^{-1}]	$k_{dissociation}$ [s^{-1}]	E_{act} [kJ/mol]
S12	7.25E+06 ± 6.02E+04		3.38E+01 ± 2.06E-02
S13	1.90E+08 ± 1.77E+07		2.58E+01 ± 2.31E-01
S14	6.10E+08 ± 6.77E+07		2.29E+01 ± 2.75E-01
S15	1.15E+09 ± 1.75E+08		2.13E+01 ± 3.78E-01
S16	1.22E+09 ± 1.20E+08		2.12E+01 ± 2.44E-01
S21		7.52E-02 ± 6.28E-04	7.94E+01 ± 2.07E-02
S23	5.72E+06 ± 1.23E+05		3.44E+01 ± 5.34E-02
S24	2.99E+08 ± 6.45E+07		2.46E+01 ± 5.35E-01
S25	1.01E+09 ± 3.49E+08		2.16E+01 ± 8.55E-01
S31		3.72E-02 ± 6.77E-03	8.11E+01 ± 4.51E-01
S32		6.81E-02 ± 1.33E-03	7.96E+01 ± 4.82E-02
S34	8.11E+06 ± 3.08E+05		3.36E+01 ± 9.42E-02
S43		9.27E-02 ± 2.92E-03	7.89E+01 ± 7.81E-02
S45	1.14E+07 ± 7.03E+05		3.27E+01 ± 1.52E-01
S54		1.36E-01 ± 6.52E-03	7.79E+01 ± 1.19E-01
S56	1.27E+07 ± 1.15E+06		3.24E+01 ± 2.24E-01
S65		1.33E-01 ± 1.09E-02	7.80E+01 ± 2.03E-01

Supplementary Table 12: All extracted rate constants and corresponding activation energies for 10 nM HI[655]. $k_{association}$ [s^{-1} M^{-1}] is the association rate constant, while $k_{dissociation}$ [s^{-1}] is the dissociation rate constant. The activation energy E_{act} [kJ/mol] is calculated as shown in eq. 3.

10 nM HI^{655} + 100 µM Zn^{2+}			
Transition	$k_{association}$ [s^{-1} M^{-1}]	$k_{dissociation}$ [s^{-1}]	E_{act} [kJ/mol]
S12	7.12E+06 ± 5.82E+04		3.39E+01 ± 2.03E-02
S13	4.16E+08 ± 6.14E+07		2.38E+01 ± 3.65E-01
S15	2.15E+10 ± 1.17E+10		1.40E+01 ± 1.35E+00
S16	9.89E+09 ± 3.40E+09		1.60E+01 ± 8.53E-01
S21		7.90E-02 ± 6.64E-04	7.93E+01 ± 2.08E-02
S23	6.73E+06 ± 1.16E+05		3.40E+01 ± 4.28E-02
S24	1.04E+09 ± 3.89E+08		2.15E+01 ± 9.27E-01
S31		3.84E-02 ± 7.46E-03	8.11E+01 ± 4.82E-01
S32		8.89E-02 ± 1.50E-03	7.90E+01 ± 4.18E-02
S34	9.73E+06 ± 2.48E+05		3.31E+01 ± 6.32E-02
S43		1.34E-01 ± 3.36E-03	7.80E+01 ± 6.20E-02
S45	1.62E+07 ± 5.87E+05		3.19E+01 ± 8.98E-02
S46	2.10E+09 ± 1.20E+09		1.98E+01 ± 1.42E+00
S54		1.80E-01 ± 6.44E-03	7.72E+01 ± 8.85E-02
S56	2.13E+07 ± 1.16E+06		3.12E+01 ± 1.35E-01
S65		1.09E-01 ± 5.92E-03	7.85E+01 ± 1.34E-01

Supplementary Table 13: All extracted rate constants and corresponding activation energies for 10 nM HI^{655} + 100 µM Zn^{2+}. $k_{association}$ [s^{-1} M^{-1}] is the association rate constant, while $k_{dissociation}$ [s^{-1}] is the dissociation rate constant. The activation energy E_{act} [kJ/mol] is calculated as shown in eq. 3.

10 nM HI^{655} + 25 µM phenol			
Transition	$k_{association}$ [s^{-1} M^{-1}]	$k_{dissociation}$ [s^{-1}]	E_{act} [kJ/mol]
S12	7.40E+06 ± 7.68E+04		3.38E+01 ± 2.57E-02
S13	1.80E+08 ± 3.75E+07		2.59E+01 ± 5.16E-01
S14	1.39E+09 ± 1.86E+08		2.08E+01 ± 3.31E-01
S21		8.39E-02 ± 6.11E-04	7.91E+01 ± 1.81E-02
S23	7.19E+06 ± 1.07E+05		3.39E+01 ± 3.69E-02
S24	2.40E+08 ± 8.06E+07		2.52E+01 ± 8.31E-01
S31		4.75E-02 ± 9.00E-03	8.05E+01 ± 4.70E-01
S32		7.38E-02 ± 9.16E-04	7.94E+01 ± 3.07E-02
S34	8.58E+06 ± 1.77E+05		3.34E+01 ± 5.13E-02
S42		2.13E-01 ± 6.72E-02	7.68E+01 ± 7.80E-01
S43		1.07E-01 ± 2.00E-03	7.85E+01 ± 4.63E-02
S45	1.21E+07 ± 3.61E+05		3.26E+01 ± 7.37E-02
S54		1.46E-01 ± 4.11E-03	7.78E+01 ± 6.99E-02
S56	1.55E+07 ± 6.75E+05		3.20E+01 ± 1.08E-01
S65		1.04E-01 ± 4.46E-03	7.86E+01 ± 1.06E-01

Supplementary Table 14: All extracted rate constants and corresponding activation energies for 10 nM HI^{655} + 25 µM phenol. $k_{association}$ [s^{-1} M^{-1}] is the association rate constant, while $k_{dissociation}$ [s^{-1}] is the dissociation rate constant. The activation energy E_{act} [kJ/mol] is calculated as shown in eq. 3

10 nM HI^{655} + 100 µM Zn^{2+} + 25 µM phenol			
Transition	$k_{association}$ [s^{-1} M^{-1}]	$k_{dissociation}$ [s^{-1}]	E_{act} [kJ/mol]
S12	7.67E+06 ± 5.23E+04		3.37E+01 ± 1.68E-02
S13	2.52E+08 ± 8.52E+07		2.51E+01 ± 8.38E-02
S14	1.25E+09 ± 8.83E+07		2.11E+01 ± 1.75E-01
S15	1.63E+09 ± 1.64E+08		2.04E+01 ± 2.49E-01
S16	1.89E+09 ± 2.10E+08		2.01E+01 ± 2.75E-01
S21		1.04E-01 ± 7.07E-04	7.86E+01 ± 1.69E-02
S23	8.92E+06 ± 7.41E+04		3.33E+01 ± 2.06E-02
S24	3.98E+08 ± 4.21E+07		2.39E+01 ± 2.62E-01
S25	2.29E+09 ± 3.94E+08		1.97E+01 ± 4.44E-01
S26	4.27E+09 ± 7.42E+08		1.80E+01 ± 4.31E-01
S31		7.83E-02 ± 1.04E-02	7.93E+01 ± 3.30E-01
S32		1.06E-02 ± 8.51E-04	7.86E+01 ± 1.99E-02
S34	1.06E+06 ± 1.02E+05		3.29E+01 ± 2.38E-02
S35	7.42E+08 ± 1.98E+08		2.24E+01 ± 6.59E-01
S36	3.19E+09 ± 8.22E+08		1.88E+01 ± 6.37E-01
S41		1.31E-01 ± 2.78E-02	7.80E+01 ± 5.27E-01
S42		2.23E-01 ± 5.74E-02	7.67E+01 ± 6.38E-01
S43		1.48E-01 ± 1.38E-03	7.77E+01 ± 2.31E-02
S45	1.50E+07 ± 1.82E+05		3.20E+01 ± 3.01E-02
S46	1.75E+09 ± 1.68E+08		2.02E+01 ± 6.61E-01
S53		1.55E-01 ± 5.45E-02	7.76E+01 ± 8.69E-01
S54		1.82E-01 ± 2.16E-03	7.72E+01 ± 2.94E-02

S56	1.88E+07 ± 3.21E+05		3.15E+01 ± 4.23E-02
S62		8.85E-02 ± 3.56E-02	7.89E+01 ± 9.96E-01
S65		1.18E-01 ± 2.01E-03	7.83E+01 ± 4.22E-02

Supplementary Table 15: All extracted rate constants and corresponding activation energies for 10 nM HI^{655} + 100 µM Zn^{2+} + 25 µM phenol. $k_{association}$ [s^{-1} M^{-1}] is the association rate constant, while $k_{dissociation}$ [s^{-1}] is the dissociation rate constant. The activation energy E_{act} [kJ/mol] is calculated as shown in eq. 3.

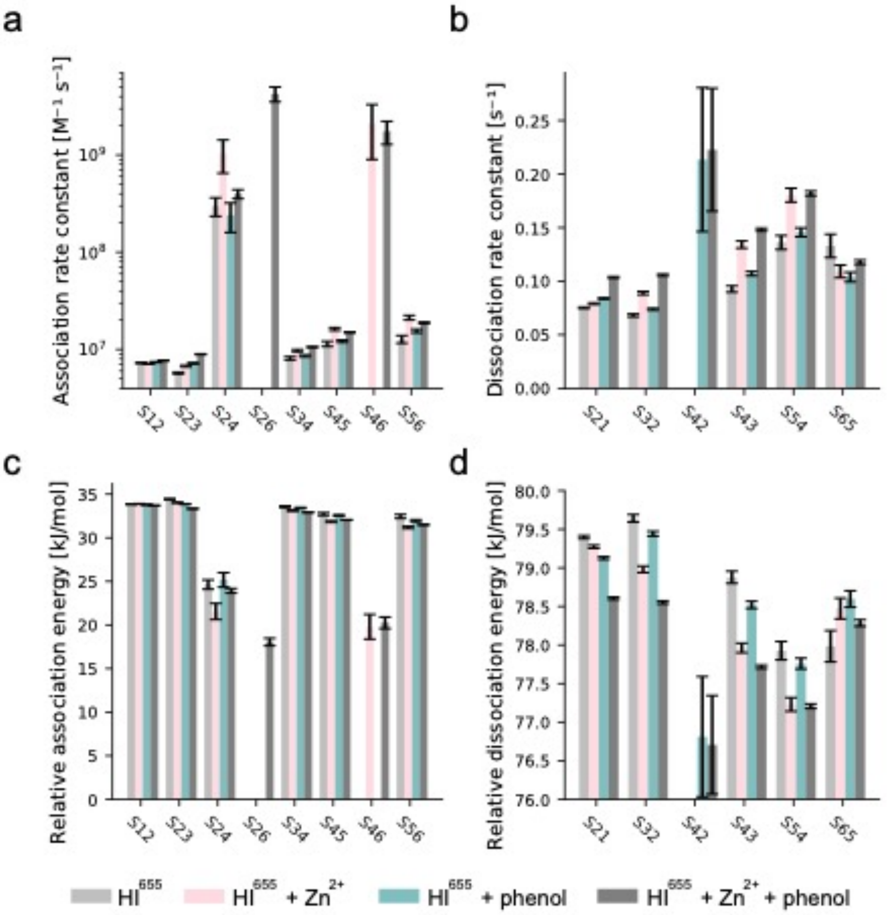

Supplementary Figure 29: Rate constants and relative activation energy for association and dissociation from HMM analysis. **a,** Rate constants for monomer addition and dimer addition for 10 nM HI^{655}, 10 nM HI^{655} + 25 µM phenol, 10 nM HI^{655} + 100 µM Zn^{2+} and 10 nM HI^{655} + 100 µM Zn^{2+} + 25 µM phenol. **b,** Rate constants for monomer disassembly for 10 nM HI^{655}, 10 nM HI^{655} + 25 µM phenol, 10 nM HI^{655} + 100 µM Zn^{2+} and 10 nMHI^{655} + 100 µM Zn^{2+} + 25 µM phenol. Error Bars are fit errors. **c,** Activation energy for monomer addition and dimer addition for 10 nM HI^{655}, 10 nM HI^{655} + 25 µM phenol, 10 nM HI^{655} + 100 µM Zn^{2+} and 10 nM HI^{655} + 100 µM Zn^{2+} + 25 µM phenol. **d,** Activation energy for monomer disassembly for 10 nM HI^{655}, 10 nM HI^{655} + 25 µM phenol, 10 nM HI^{655} + 100 µM Zn^{2+} and 10 nM HI^{655} + 100 µM Zn^{2+} + 25 µM phenol. Error bars are fit errors.

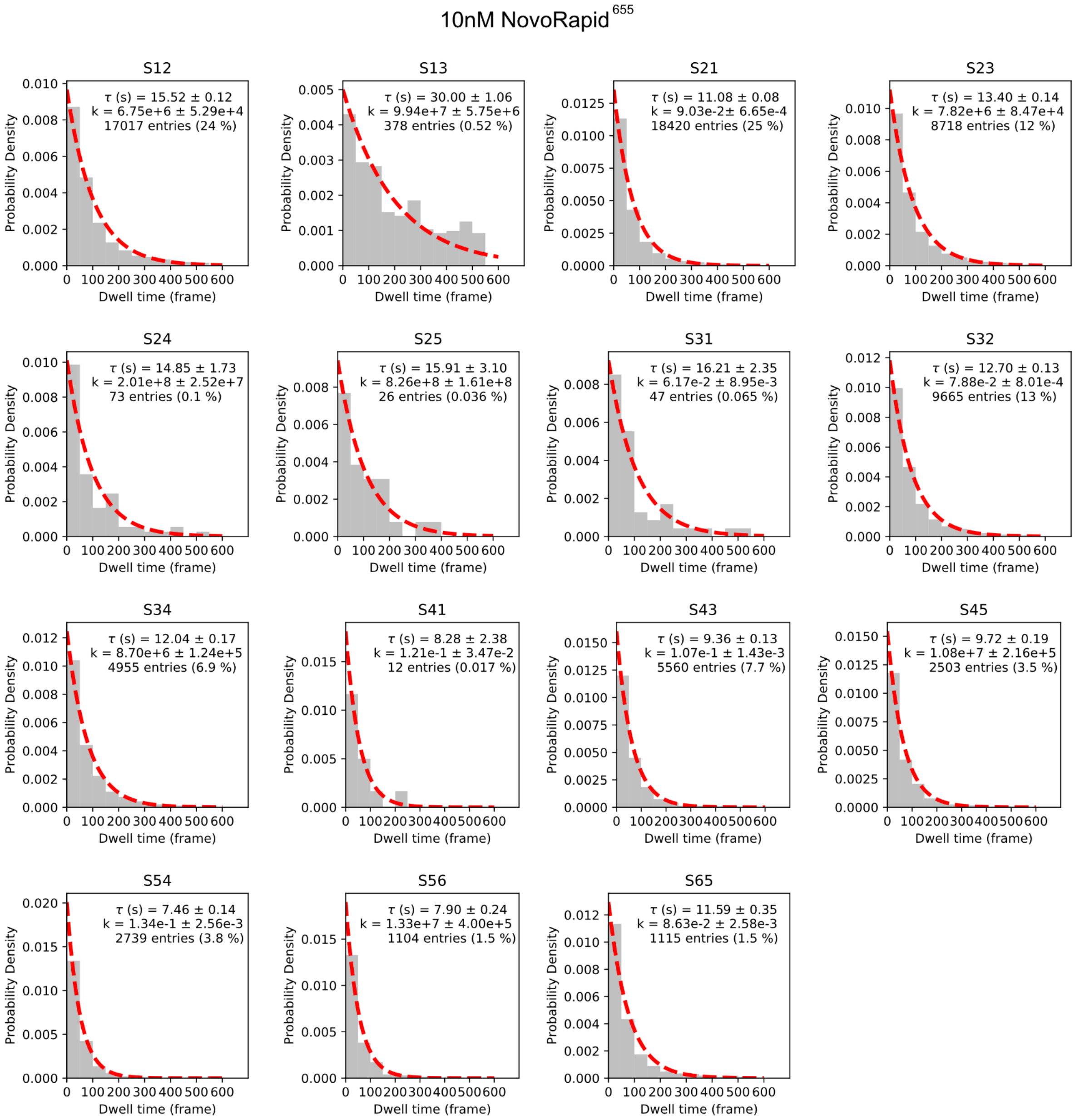

Supplementary Figure 30: Single exponential fit (red dotted line) to dwell times (gray) for 17 separable clusters of unique transitions for 10 nM NovoRapid^{655}. Within each fit is the extracted overall dwell time, rate constant and density (how many times the transition is observed) shown.

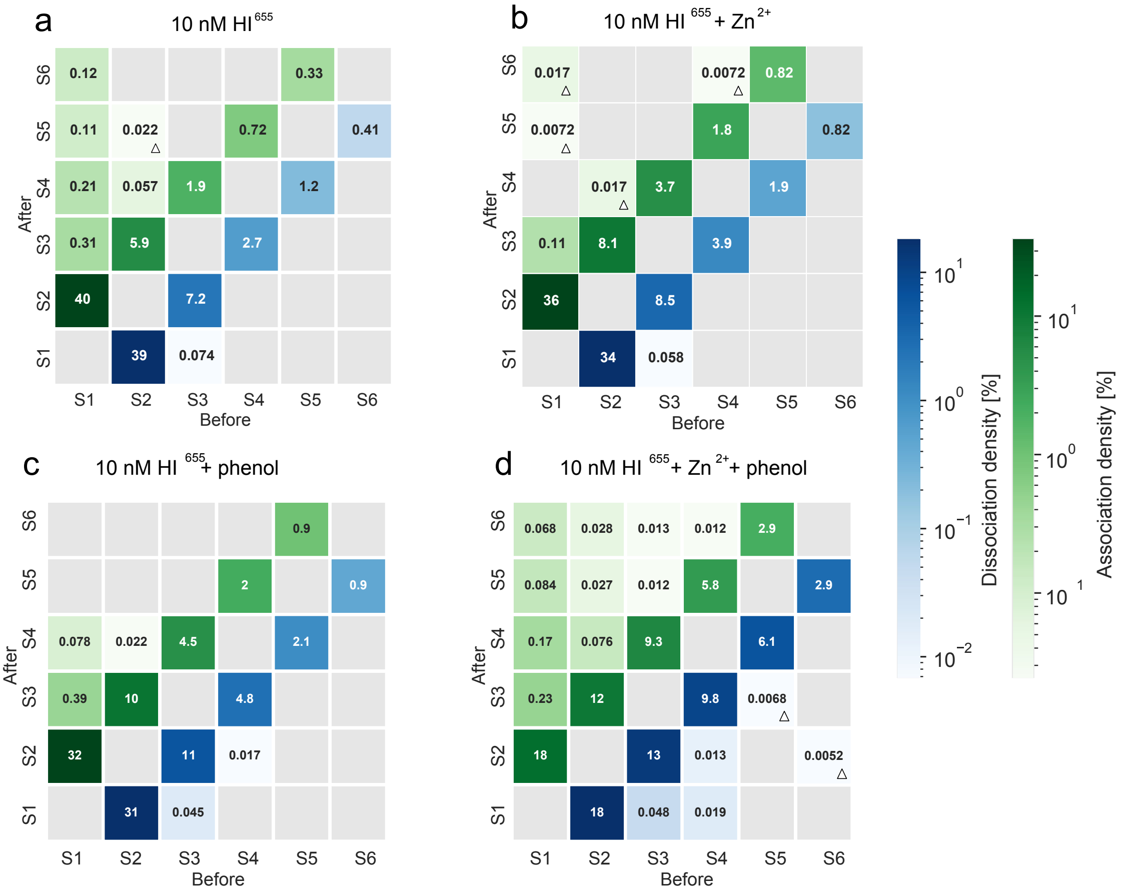

Supplementary Figure 31: CHESS (Complete HEatmap of State transitionS) plot shows thermodynamic parameters for each separate transition from a before state (x-axis) to an after state (y-axis). The numbers within the squares are the value for transition density. Gray squares are transitions with no data points. Transitions involving S0 (background) have been removed. Triangle denotes if less than 10 transitions were observed. Extracted association and dissociation transition densities for each transition for **a,** 10 nM HI^{655}, **b,** 10 nM HI^{655} + 100 μM Zn^{2+}, **c,** 10 nM HI^{655} + 25 μM phenol and **d,** 10 nM HI^{655} + 100 μM Zn^{2+} + 25 μM phenol.

	HI^{655}	HI^{655} + Zn^{2+}	HI^{655} + phenol	HI^{655} + Zn^{2+} + phenol
n_h	0.54 ± 0.04	0.59 ± 0.05	0.57 ± 0.04	0.59 ± 0.04
K	2.61 ± 0.40	0.87 ± 0.14	1.50 ± 0.25	0.18 ± 0.02
B_{max}	0.86 ± 0.01	0.92 ± 0.02	0.90 ± 0.02	0.97 ± 0.01

Supplementary Table 16: Fitting parameters from fitting fraction of hexameric oligomers with a Hill equation.

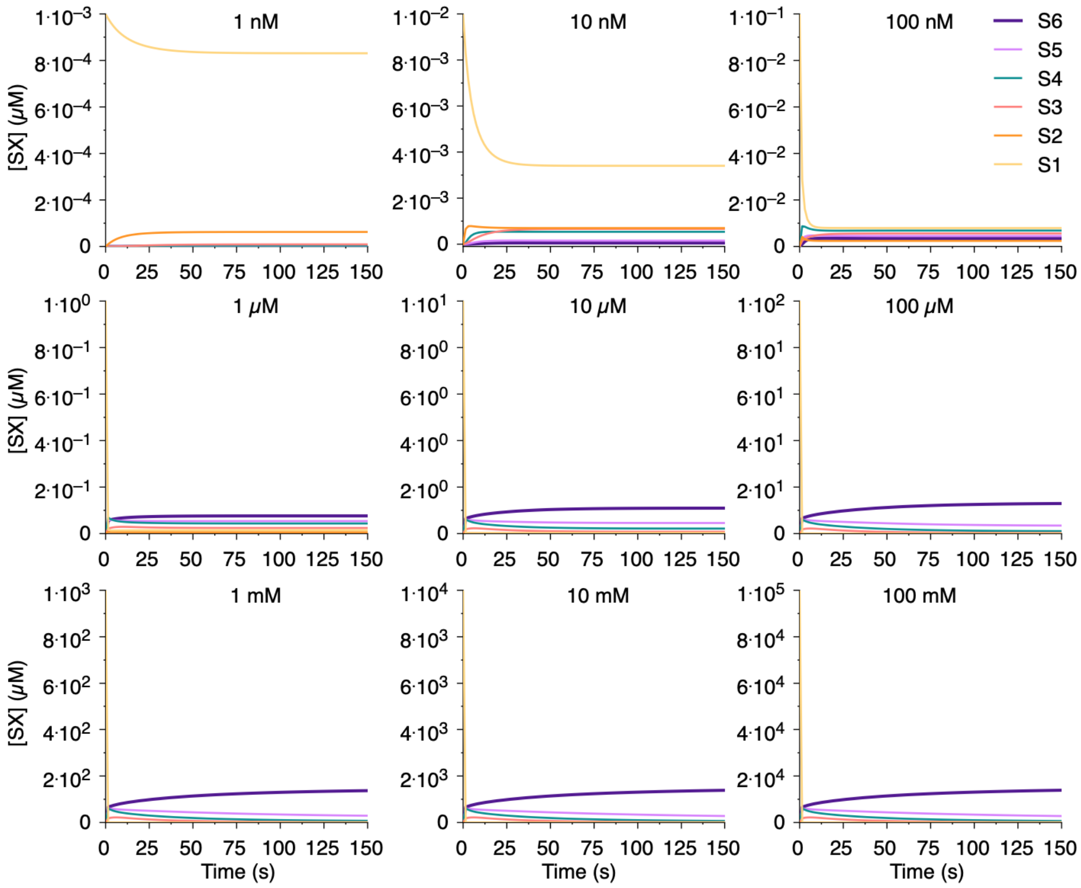

Supplementary Figure 32: Simulations for HI[655] with insulin concentration ranging from 1 nM to 100 mM.

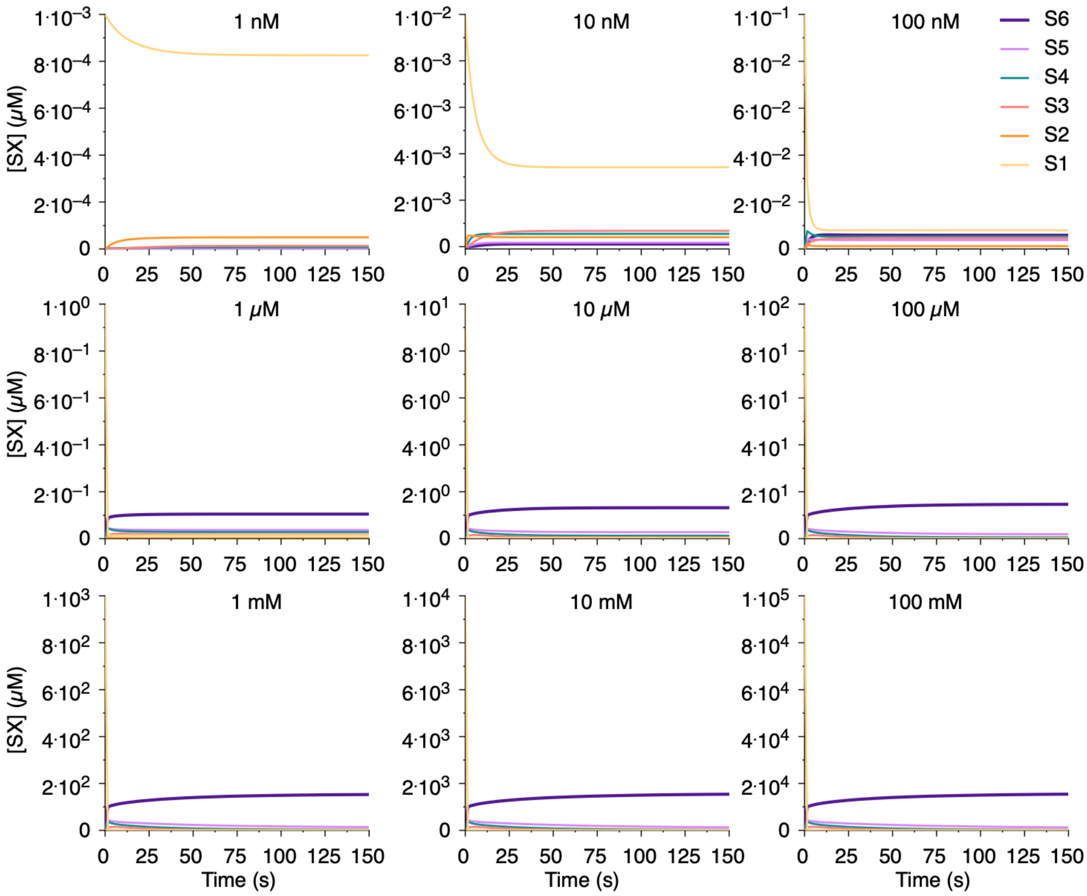

Supplementary Figure 33: Simulations for HI^{655} + 100 µM Zn^{2+} with insulin concentration ranging from 1 nM to 100 mM.

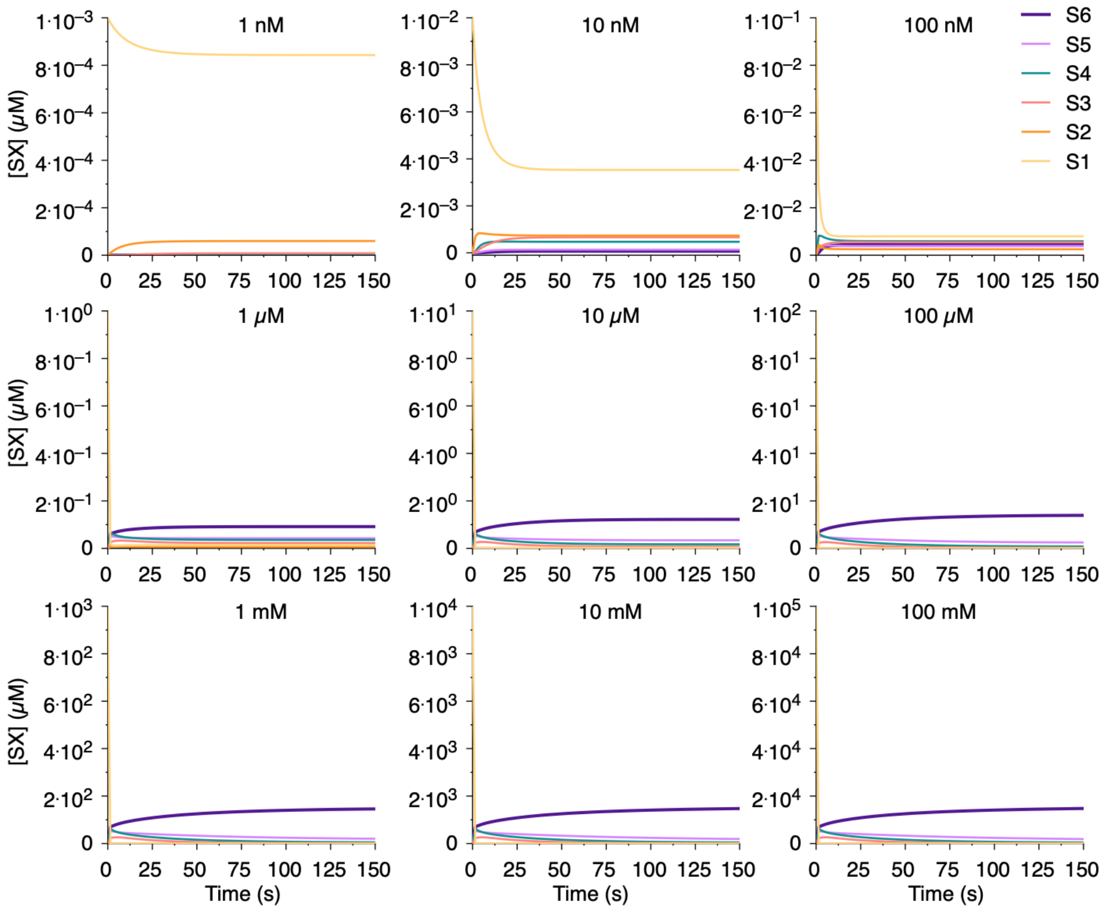

Supplementary Figure 34: Simulations for HI[655] + 25 μM phenol with insulin concentration ranging from 1 nM to 100 mM.

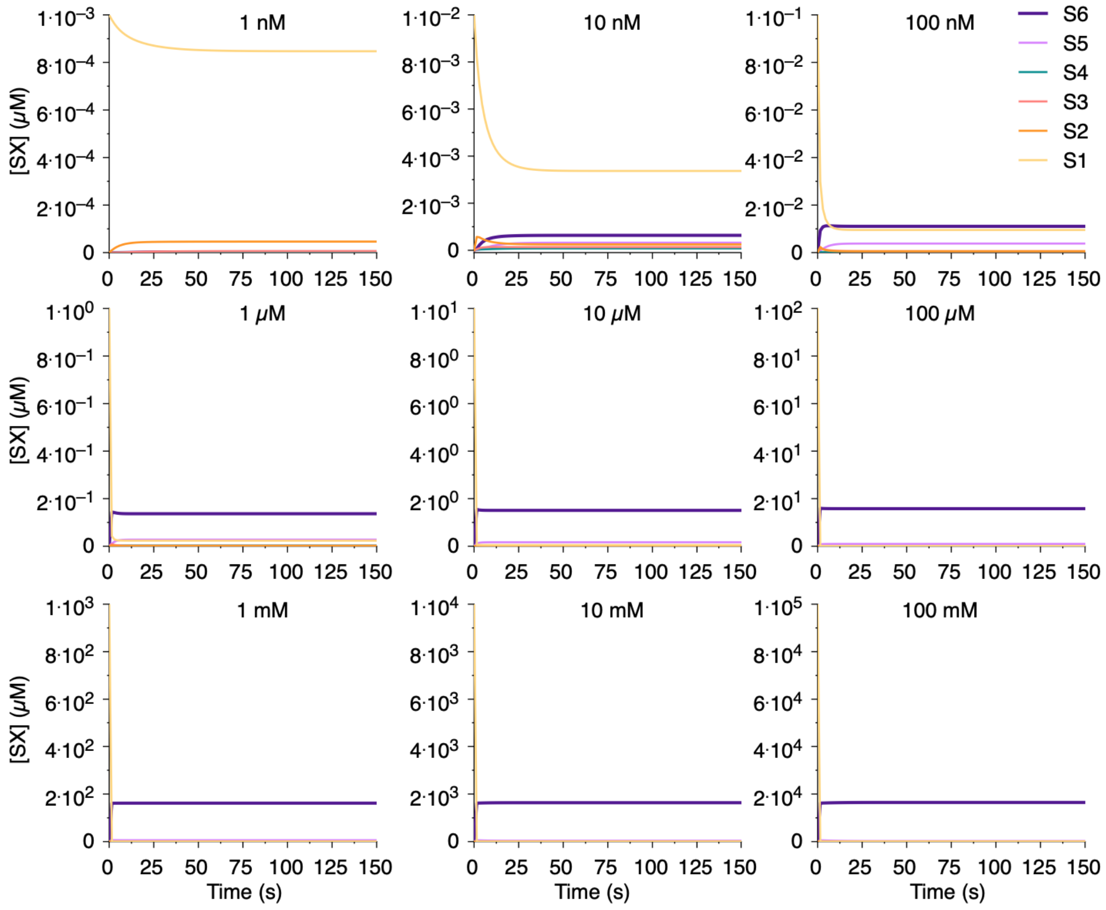

Supplementary Figure 35: Simulations for HI[655] + 25 μM phenol + 100 μM Zn^{2+} with insulin concentration ranging from 1 nM to 100 mM.

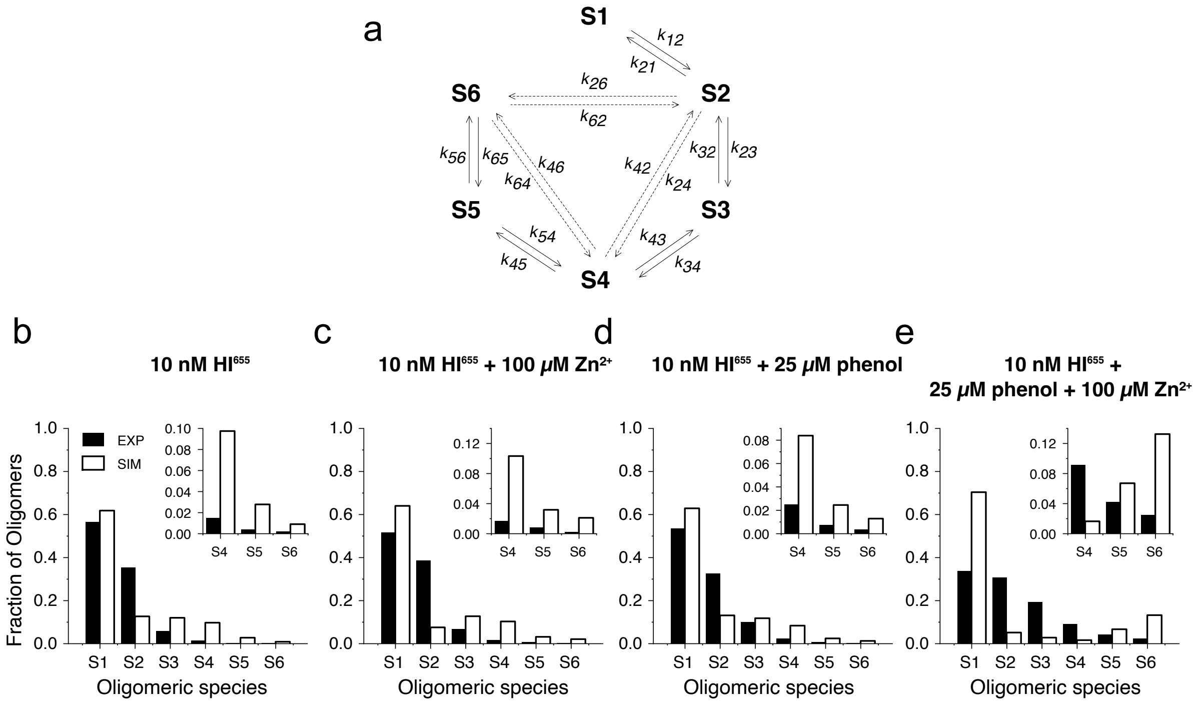

Supplementary Figure 36: Simulations **a,** Model used for simulating time evolution of oligomeric species including monomer and dimer association and dissociation steps. Comparison of experimental (black bars) and experimentally (empty bars) found fraction of oligomers for **b,** 10 nM HI^{655}, **c,** 10 nM HI^{655} + 100 μM Zn^{2+}, **d,** 10 nM HI^{655} + 25 μM phenol and **e,** 10 nM HI^{655} + phenol + Zn^{2+}. Simulated oligomeric fractions have no associated error. Error bars representing the standard error from the experimental oligomeric fractions are smaller than the lines.

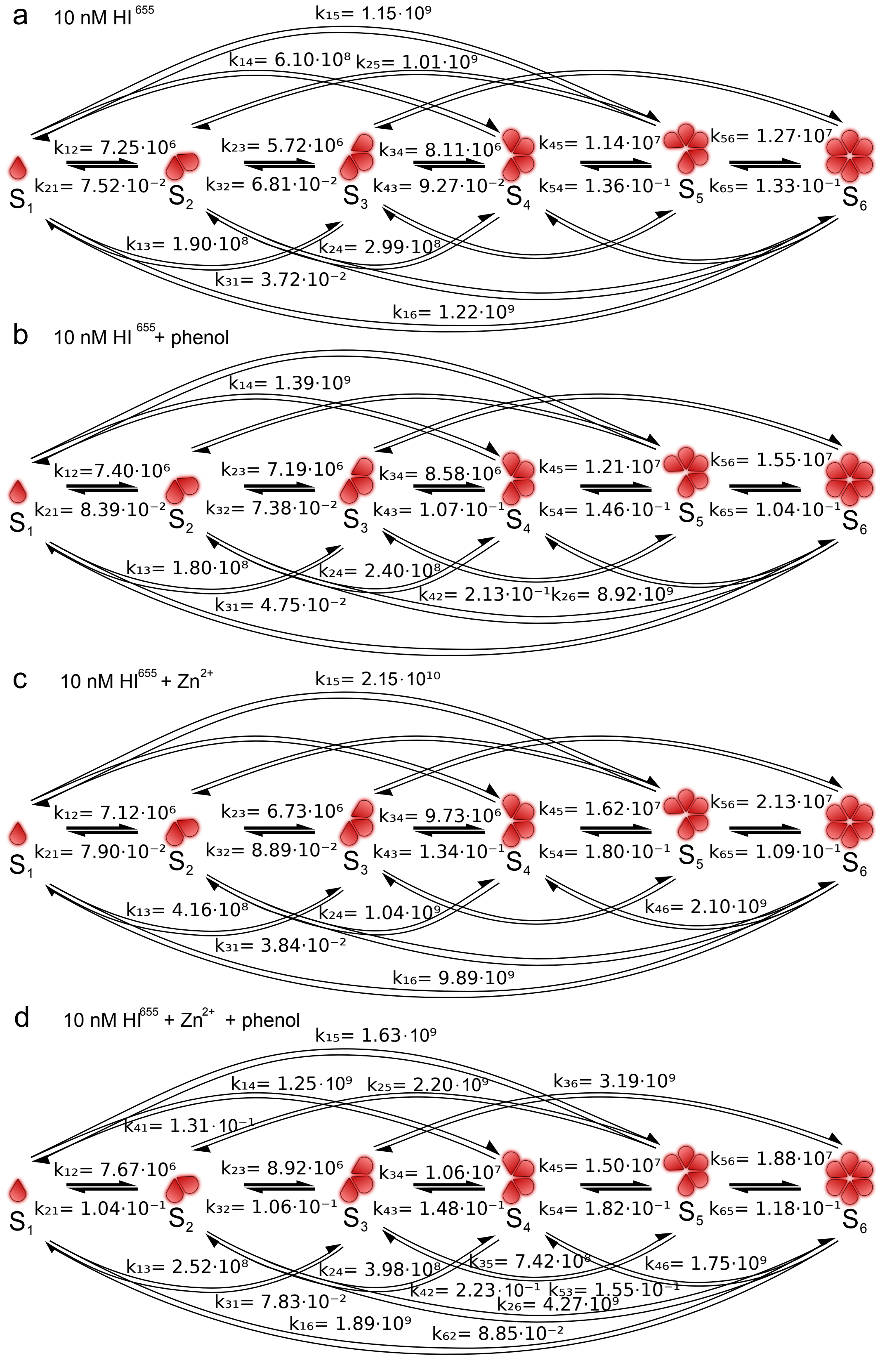
a
10 nM HI^{655}
$k_{15}= 1.15\cdot10^{9}$
$k_{14}= 6.10\cdot10^{8}$
$k_{25}= 1.01\cdot10^{9}$
$k_{12}= 7.25\cdot10^{6}$
$k_{21}= 7.52\cdot10^{-2}$
$k_{23}= 5.72\cdot10^{6}$
$k_{32}= 6.81\cdot10^{-2}$
$k_{34}= 8.11\cdot10^{6}$
$k_{43}= 9.27\cdot10^{-2}$
$k_{45}= 1.14\cdot10^{7}$
$k_{54}= 1.36\cdot10^{-1}$
$k_{56}= 1.27\cdot10^{7}$
$k_{65}= 1.33\cdot10^{-1}$
S_1 S_2 S_3 S_4 S_5 S_6
$k_{13}= 1.90\cdot10^{8}$
$k_{24}= 2.99\cdot10^{8}$
$k_{31}= 3.72\cdot10^{-2}$
$k_{16}= 1.22\cdot10^{9}$
b
10 nM HI^{655}+ phenol
$k_{14}= 1.39\cdot10^{9}$
$k_{12}=7.40\cdot10^{6}$
$k_{21}= 8.39\cdot10^{-2}$
$k_{23}= 7.19\cdot10^{6}$
$k_{32}= 7.38\cdot10^{-2}$
$k_{34}= 8.58\cdot10^{6}$
$k_{43}= 1.07\cdot10^{-1}$
$k_{45}= 1.21\cdot10^{7}$
$k_{54}= 1.46\cdot10^{-1}$
$k_{56}= 1.55\cdot10^{7}$
$k_{65}= 1.04\cdot10^{-1}$
S_1 S_2 S_3 S_4 S_5 S_6
$k_{13}= 1.80\cdot10^{8}$
$k_{24}= 2.40\cdot10^{8}$
$k_{42}= 2.13\cdot10^{-1}$
$k_{26}= 8.92\cdot10^{9}$
$k_{31}= 4.75\cdot10^{-2}$
c
10 nM HI^{655} + Zn^{2+}
$k_{15}= 2.15\cdot10^{10}$
$k_{12}= 7.12\cdot10^{6}$
$k_{21}= 7.90\cdot10^{-2}$
$k_{23}= 6.73\cdot10^{6}$
$k_{32}= 8.89\cdot10^{-2}$
$k_{34}= 9.73\cdot10^{6}$
$k_{43}= 1.34\cdot10^{-1}$
$k_{45}= 1.62\cdot10^{7}$
$k_{54}= 1.80\cdot10^{-1}$
$k_{56}= 2.13\cdot10^{7}$
$k_{65}= 1.09\cdot10^{-1}$
S_1 S_2 S_3 S_4 S_5 S_6
$k_{13}= 4.16\cdot10^{8}$
$k_{24}= 1.04\cdot10^{9}$
$k_{46}= 2.10\cdot10^{9}$
$k_{31}= 3.84\cdot10^{-2}$
$k_{16}= 9.89\cdot10^{9}$
d
10 nM HI^{655} + Zn^{2+} + phenol
$k_{15}= 1.63\cdot10^{9}$
$k_{14}= 1.25\cdot10^{9}$
$k_{25}= 2.20\cdot10^{9}$
$k_{36}= 3.19\cdot10^{9}$
$k_{41}= 1.31\cdot10^{-1}$
$k_{12}= 7.67\cdot10^{6}$
$k_{21}= 1.04\cdot10^{-1}$
$k_{23}= 8.92\cdot10^{6}$
$k_{32}= 1.06\cdot10^{-1}$
$k_{34}= 1.06\cdot10^{7}$
$k_{43}= 1.48\cdot10^{-1}$
$k_{45}= 1.50\cdot10^{7}$
$k_{54}= 1.82\cdot10^{-1}$
$k_{56}= 1.88\cdot10^{7}$
$k_{65}= 1.18\cdot10^{-1}$
S_1 S_2 S_3 S_4 S_5 S_6
$k_{13}= 2.52\cdot10^{8}$
$k_{24}= 3.98\cdot10^{8}$
$k_{35}= 7.42\cdot10^{8}$
$k_{46}= 1.75\cdot10^{9}$
$k_{42}= 2.23\cdot10^{-1}$
$k_{53}= 1.55\cdot10^{-1}$
$k_{31}= 7.83\cdot10^{-2}$
$k_{26}= 4.27\cdot10^{9}$
$k_{16}= 1.89\cdot10^{9}$
$k_{62}= 8.85\cdot10^{-2}$

Supplementary Figure 37: Proposed mechanistic model for insulin oligomerization. Rate constants are obtained from Hidden Markov Model analysis for **a,** 10 nM HI^{655}, **b,** 10 nM HI^{655} + 25 µM phenol, **c,** 10 nM HI^{655} + 100 µM Zn^{2+} and **d,** 10 nM HI^{655} + 100 µM Zn^{2+} + 25 µM phenol and plotted into a cartoon of the insulin oligomerization mechanism. The found rate constants suggest that oligomerization mostly happens via monomeric addition of insulin, while addition of Zn^{2+} promotes dimeric insulin addition via monomer - dimer - tetramer - hexamer equilibrium and Zn^{2+} and phenol reroutes the pathway to monomer - dimer - hexamer equilibrium.

10 nM HI^{655}		
Transition	$k_{association}$ [s^{-1} M^{-1}]	$k_{dissociation}$ [s^{-1}]
S12	7.25E+06 ± 6.02E+04	
S21		7.52E-02 ± 6.28E-04
S23	5.72E+06 ± 1.23E+05	
S32		6.81E-02 ± 1.33E-03
S34	8.11E+06 ± 3.08E+05	
S43		9.27E-02 ± 2.92E-03
S45	1.14E+07 ± 7.03E+05	
S54		1.36E-01 ± 6.52E-03
S56	1.27E+07 ± 1.15E+06	
S65		1.33E-01 ± 1.09E-02
S24	2.99E+08 ± 6.45E+07	
S42		2.13E-01 ± 6.72E-02
S26	0.00E ± 0.00	
S62		0.00E ± 0.00
S46	0.00E ± 0.00	
S64		0.01

Supplementary Table 17: Rate constants for dissociation and association used for the simulations with HI^{655}. Values in black are experimentally found as described earlier. Blue value is the experimentally found value for HI^{655} + 25 µM phenol, as this dissociation rate constant was unobtainable from HI^{655} experiments. S64 (red) was set as an arbitrary upper limit for the dissociation of hexamer into tetramer.

10 nM HI^{655}+ 100 μM Zn^{2+}		
Transition	$k_{association}$ [s^{-1} M^{-1}]	$k_{dissociation}$ [s^{-1}]
S12	7.12E+06 ± 5.82E+04	
S21		7.90E-02 ± 6.64E-04
S23	6.73E+06 ± 1.16E+05	
S32		8.89E-02 ± 1.50E-03
S34	9.73E+06 ± 2.48E+05	
S43		1.34E-01 ± 3.36E-03
S45	1.62E+07 ± 5.87E+05	
S54		1.80E-01 ± 6.44E-03
S56	2.13E+07 ± 1.16E+06	
S65		1.09E-01 ± 5.92E-03
S24	1.04E+09 ± 3.89E+08	
S42		2.13E-01 ± 6.72E-02
S26	0.00E ± 0.00	
S62		0.00E ± 0.00
S46	2.10E+09 ± 1.20E+09	
S64		0.01

Supplementary Table 18: Rate constants for dissociation and association used for the simulations with HI^{655} + 100 μM Zn^{2+}. Values in black are experimentally found as described earlier. Blue value is the experimentally found value for HI^{655} + 25 μM phenol, as this dissociation rate constant was unobtainable from HI^{655} experiments. S64 (red) was set as an arbitrary upper limit for the dissociation of hexamer into tetramer.

10 nM HI^{655} + 25 µM phenol		
Transition	$k_{association}$ [s^{-1} M^{-1}]	$k_{dissociation}$ [s^{-1}]
S12	7.40E+06 ± 7.68E+04	
S21		8.39E-02 ± 6.11E-04
S23	7.19E+06 ± 1.07E+05	
S32		7.38E-02 ± 9.16E-04
S34	8.58E+06 ± 1.77E+05	
S43		1.07E-01 ± 2.00E-03
S45	1.21E+07 ± 3.61E+05	
S54		1.46E-01 ± 4.11E-03
S56	1.55E+07 ± 6.75E+05	
S65		1.04E-01 ± 4.46E-03
S24	2.40E+08 ± 8.06E+07	
S42		2.13E-01 ± 6.72E-02
S26	0.00E ± 0.00	
S62		0.00E ± 0.00
S46	0.00E ± 0.00	
S64		0.01

Supplementary Table 19: Rate constants for dissociation and association used for the simulations with HI^{655} + 25 µM phenol. Values in black are experimentally found as described earlier. S64 (red) was set as an arbitrary upper limit for the dissociation of hexamer into tetramer.

10 nM HI^{655} + 25 µM phenol + 100 µM Zn^{2+}		
Transition	$k_{association}$ [s^{-1} M^{-1}]	$k_{dissociation}$ [s^{-1}]
S12	7.67E+06 ± 5.23E+04	
S21		1.04E-01 ± 7.07E-04
S23	8.92E+06 ± 7.41E+04	
S32		1.06E-02 ± 8.51E-04
S34	1.06E+07 ± 1.02E+05	
S43		1.48E-01 ± 1.38E-03
S45	1.50E+07 ± 1.82E+05	
S54		1.82E-01 ± 2.16E-03
S56	1.88E+07 ± 3.21E+05	
S65		1.18E-01 ± 2.01E-03
S24	3.98E+08 ± 4.21E+07	
S42		2.23E-01 ± 5.74E-02
S26	4.27E+09 ± 7.42E+08	
S62		8.85E-02 ± 3.56E-02
S46	1.75E+09 ± 4.68E+08	
S64		0.01

Supplementary Table 20: Rate constants for dissociation and association used for the simulations with HI^{655} + 25 µM phenol + 100 µM Zn^{2+} . Values in black are experimentally found as described earlier. S64 (red) was set as an arbitrary upper limit for the dissociation of hexamer into tetramer.
