# Supplementary material for: Enhanced hexamerization of insulin via assembly pathway rerouting revealed by single particle studies
Source: Commun Biol. 2023 Feb 15;6:178. doi: 10.1038/s42003-022-04386-6 (PMC9932072; doi:10.1038/s42003-022-04386-6)
Supplement: Supplementary file 3 — Description of Additional Supplementary Files [file 42003_2022_4386_MOESM3_ESM.pdf]

Description of Additional Supplementary Files

File name: Supplementary Data 1

Description: Numerical data of fig 1f

File name: Supplementary Data 2

Description: Numerical data of fig 2a

File name: Supplementary Data 3

Description: Numerical data of fig 2b

File name: Supplementary Data 4

Description: Numerical data of fig 2c

File name: Supplementary Data 5

Description: Numerical data of fig 2d

File name: Supplementary Data 6

Description: Numerical data of fig 4a

File name: Supplementary Data 7

Description: Numerical data of fig 4b

File name: Supplementary Data 8

Description: Numerical data of fig 4c

File name: Supplementary Data 9

Description: Numerical data of fig 4d

File name: Supplementary Data 10

Description: Numerical data of fig 5a-d

File name: Supplementary Data 11

Description: Numerical data of fig 5e
